# Clinical and genetic studies for a cohort of patients with congenital stationary night blindness

**DOI:** 10.1186/s13023-024-03091-3

**Published:** 2024-03-06

**Authors:** Lijuan Huang, Xueqing Bai, Yan Xie, Yunyu Zhou, Jin Wu, Ningdong Li

**Affiliations:** 1 Department of Ophthalmology, The Second Affiliated Hospital of Fujian Medical University, Quanzhou, 362000 China; 2 Department of Ophthalmology, Beijing Children’s Hospital, Capital Medical University, Beijing, 100045 China; 3 Department of Ophthalmology, Shanghai General Hospital, Shanghai, 200940 China

**Keywords:** Congenital stationary night blindness, Myopia, Strabismus, Mutation

## Abstract

**Background:**

Congenital stationary night blindness (CSNB) is an inherited retinal disorder. Most of patients have myopia. This study aims to describe the clinical and genetic characteristics of fifty-nine patients with CSNB and investigate myopic progression under genetic cause.

**Results:**

Sixty-five variants were detected in the 59 CSNB patients, including 32 novel and 33 reported variants. The most frequently involved genes were *NYX, CACNA1F*, and *TRPM1*. Myopia (96.61%, 57/59) was the most common clinical finding, followed by nystagmus (62.71%, 37/59), strabismus (52.54%, 31/59), and nyctalopia (49.15%, 29/59). An average SE of -7.73 ± 3.37 D progressed to -9.14 ± 2.09 D in *NYX* patients with myopia, from − 2.24 ± 1.53 D to -4.42 ± 1.43 D in those with *CACNA1F*, and from − 5.21 ± 2.89 D to -9.24 ± 3.16 D in those with *TRPM1* during the 3-year follow-up; the *TRPM1* group showed the most rapid progression.

**Conclusions:**

High myopia and strabismus are distinct clinical features of CSNB that are helpful for diagnosis. The novel variants identified in this study will further expand the knowledge of variants in CSNB and help explore the molecular mechanisms of CSNB.

**Supplementary Information:**

The online version contains supplementary material available at 10.1186/s13023-024-03091-3.

## Introduction

Congenital stationary night blindness (CSNB) refers to a group of inherited retinal disorders caused by defective signal processing from photoreceptor cells to the bipolar cells in the retina. It is generally characterized by impaired night vision, decreased visual acuity, nystagmus, myopia, and strabismus [1]. Based on the electroretinography (ERG) recordings, CSNB can be classified into two groups of the Schubert-Bornschein type and Riggs type [2]. The Schubert-Bornschein type is characterized by a selectively reduced ERG b-wave at the scotopic light flash, indicating dysfunction at the level of bipolar cells. The Riggs type is characterized by a proportional reduction of the a- and b-waves, indicating dysfunction at the level of the photoreceptor and bipolar cells. Moreover, the Schubert-Bornschein type can be subdivided into complete and incomplete forms. The complete CSNB (cCSNB) is characterized by a severely reduced b-wave with a normal a-wave under photopic conditions indicating ON bipolar dysfunction. The incomplete CSNB (icCSNB) is characterized by a reduced rod b-wave and substantially reduced cone responses indicating dysfunction of both ON and OFF bipolar cells [3]. 

CSNB can be inherited in an autosomal dominant (AD), an autosomal recessive (AR), or an X-linked (XL) manner [4]. To date, 17 genes have been identified to be associated with CSNB. Three genes, namely *GNAT1* (OMIM*610,444), *PDE6B* (OMIM*163,500), and *RHO* (OMIM*610,445), are associated with the AD form, two genes of *CACNA1F* (OMIM*300,071) and *NYX* (OMIM*300,278) are associated with the XL form, and other 12 genes, including *CABP4* (OMIM*608,965), *GNAT1* (OMIM*616,389), *GUCY2D* (OMIM*618,555), *GNB3* (OMIM*617,024), *GPR179* (OMIM*614,515), *GRK1* (OMIM*613,411), *GRM6* (OMIM*257,270), *LRIT3* (OMIM*615,058), *RDH5* (OMIM*601,617), *SAG* (OMIM*181,031), *SLC24A1* (OMIM*613,830) and *TRPM1* (OMIM*603,576) are associated with the AR form [5, 6]. The genes of *RHO*, *GNAT1*, *PDE6B* are involved in the phototransduction cascade, and the genes of *RDH5*, *GRK1*, and *SAG* play important roles in retinoid recycling. The remaining genes are mainly involved in signaling transduction by mediating neurotransmitter release of glutamate from photoreceptors to neighboring bipolar cells. As a calcium voltage-gated channel subunit, the protein encoded by *CACNA1F* is in the presynaptic membrane between the photoreceptors and the bipolar cells and regulates calcium influx, triggering the release of glutamate from the photoreceptors to the bipolar cells. The genes of *GRM6*, *GPR179*, *NYX*, *TRPM1*, and *LRIT3* are mainly involved in glutamate-induced signal transduction postsynaptic from the photoreceptor to the bipolar cells. As a receptor for glutamate, mGluR6, encoded by the *GRM6* gene, controls the closure of the ion channel formed by TRPM1 which requires NYX to maintain its proper location on the bipolar cells. Mutations in the above genes would disturb the electrical signal transduction from the photoreceptors to the bipolar cells, resulting in CSNB [7]. 

It has been suggested that pathogenic variants in certain genes are associated with either cCSNB or iCSNB. The cCSNB form has been identified in association with mutations in the *NYX*, *GRM6*, *TRPM1*, *SLCC24A1*, *GPR179*, *LRIT3*, *GNAT1*, *GUCY2D*, *GNB3*, and *RHO* genes, and the icCSNB form is attributed to mutations in the *CACN11F*, *CABP4*, and *PDE6B* genes. Patients with cCSNB often present with early-onset myopia, decreased visual acuity, nystagmus, and strabismus. Some patients with icCSNB may have near-normal visual acuity without symptoms of myopia and/or nystagmus. Whatever the cause, environmental or genetic, most cases of myopia may increase with age [8]. Here, we present a clinical and genetic study of a cohort of Han Chinese patients with CSNB and assess their myopia progression and axis length changes over a 3-year period. We classify CSNB into the cCSNB and icCSNB types according to the ERG and disease-causing gene.

## Methods

### Participants

Medical records were retrospectively reviewed for 59 affected children with CSNB in this study. The study cohort included those patients who were diagnosed as the CSNB based on ERG, underwent molecular testing, and had ocular examination records from 3 years to 6 years old. Each patient underwent careful ocular examinations, including the best corrected visual acuity (BCVA), color vision, cycloplegic refraction, anterior segment of their eyes using a slit-lamp microscope, and fundus using direct and indirect ophthalmoscope. Fundus photography was performed using a Canon CR-2 PLUS AF Digital Non-mydriatic Retinal Camera (Canon Inc, Japan). Full-field ERG was recorded using the RETI-scan 21 system (Roland Company, Germany) according to the standards of the International Society for Clinical Electrophysiology of Vision (http://www.iscev.org). The OCT scans were obtained using a Spectralis OCT (Heidelberg Engineering GmbH, Germany). Axial length (AL) was measured using ZEISS IOL Master (Carl Zeiss Meditec, Jena, Germany). Spherical equivalent (SE) defined as the spherical power plus half of the cylinder power in diopters (D) was determined by cycloplegic refraction and streak retinoscopy. Ocular motility and ocular alignment were evaluated at the nine diagnostic gaze positions. Binocular sensory status was evaluated by the Bagolini striated glasses at near and distance, and by stereoacuity assessment at near using the Titmus Stereo Test. All data, including age, BCVA, SE, AL, ocular motor, and sensory status, were collected at each visit. Follow-up visits were scheduled every three to four months for each affected child. The study was approved by the ethics committee of Beijing Children’s Hospital ([2022]-E-213-R) and conducted in accordance with the Declaration of Helsinki. Informed consent was obtained from all participants or their guardians for the study, in accordance with the guidelines on sample collection for human genetic diseases issued by the Chinese Ministry of Public Health.

### Molecular diagnosis

Samples of 3 µg of genomic DNA were extracted from the peripheral venous blood of the patients and their family members using the Roche DNA Extraction Kit (Roche Biochemical, Inc), and fragmented with a S220 Focused-ultrasonicator (Covaris, Massachusetts, USA). The fragmented DNA samples were subjected to an exome capture procedure using an in-house designed capture panel of the GenCap Capture Kit (MyGenostics Inc, Beijing, China). The pooled libraries of the samples were sequenced using an Illumina HiSeq X Ten platform (Illumina, San Diego, CA) with 150 bp paired-end reads. Sequence reads were aligned to the Genome Reference Consortium Human Genome Build 37 (GRCh37/hg19). Variants were analyzed through the Genome Analysis Toolkit (GATK) program and only variants with a GATK-assigned quality criterion score > 50.0 were considered for downstream analyses. Variants were further annotated by the ANNOVAR software (http://annovar.openbioinformatics.org/en/latest/), which combines multiple public databases such as 1000 Genomes, ESP6500, dbSNP, gnomAD, ClinVar, and HGMD, and one commercial MyGenostics database. Copy number variants were detected using the CNV kit based on the read-depth algorithm. The pathogeneticity of the missense mutations was predicted by PolyPhen 2 (http://genetics.bwh.harvard.edu/pph2/), Sorting Intolerant from Tolerant (SIFT, https://sift.bii.a-star.edu.sg/), and Combined Annotation Dependent Depletion (CADD, https://cadd.gs.washington.edu/). Splicing effects of the variants were assessed using the SpliceAI program [9]. Only candidate variants associated with the clinical features of the patients were retained for inheritance analysis in core family members available using Sanger sequencing. The pathogenicity assessment was done according to the guidelines of the American College of Medical Genetics and Genomics (ACMG) [10]. Pathogenic variations were named following the nomenclature recommended by the Human Genomic Variation Society (HGVS).

### Statistical analysis

To investigate myopia progression, SE and AL were evaluated by mixed model repeated measures with individual subject as random effect at every 3–4 month for 3 years from the age 3 to age 6 years. All values reported as mean ± SD for variables, respectively. The mean values between two eyes of SE and AL for each participant were used in our analysis. Statistical significance was set at *p*-value < 0.05. All statistical analyses were performed by R (version, 4.1.0).

## Results

### Genetic findings

**Fig. 1:**
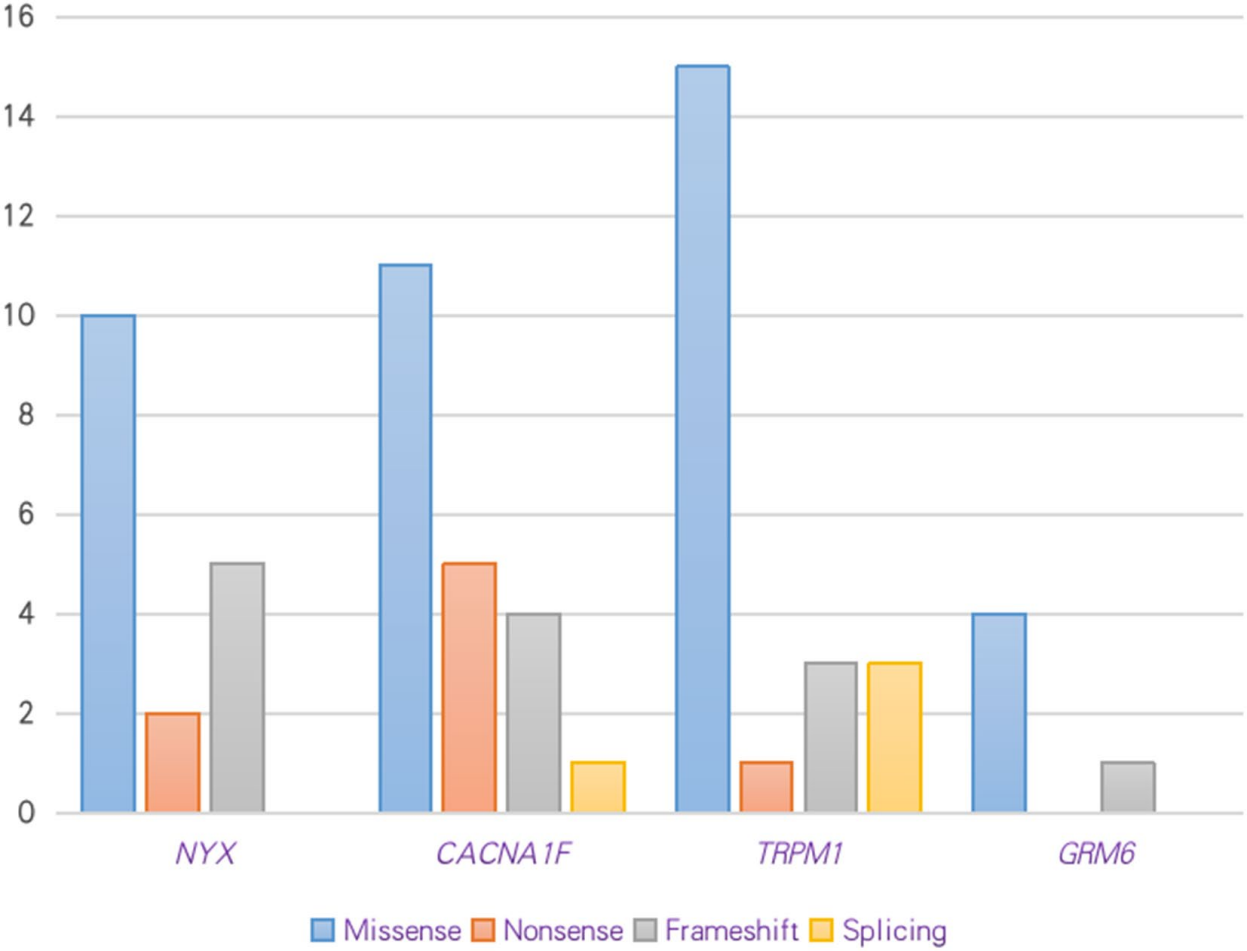
Variants type in congenital stationary night blindness patients in this study

Sixty-five variants were detected in the *NYX*, *CACNA1F*, *TRPM1* and *GRM6* genes, twenty of which were detected in *TRPM1*, followed by 22 in *CACNA1F*, 17 in *NYX*, and 6 in *GRM6* (Fig. 1). Thirty-two of 65 variants were novel identified in this study, including 8 in *NYX*, 10 in *CACNA1F*, 14 in *TRPM1*, and 3 in *GRM6*, and the remaining 33 variants were previously reported (Table 1, Supplemental Table S1). These variants included missense, nonsense, indel, and splice site defects. The calculated scores for all mutations using the programs of SIFT, Polyphen-2, CADD, and ACMG classification were listed in Supplemental Table S2. The novel identified missense variants were predicted to be deleterious to the structure of the corresponding protein, which were further confirmed by the 3-D model construction (Supplemental Pymol). Co-segregation analysis was performed on nine families with the missense variants, including four of c.553 A > C, c.611G > T, c.662T > G, and c.719 A > G in *NYX*, and five of c.1714T > C, c.2266 A > T, c.3758 C > T, c.4097T > C, and c.5429G > A in *CACNA1F*, and showed that each missense variant was co-segregated with the affected males and/or the female carriers in the families (Supplemental Pedgrees). Nonsense variants produce truncated mRNA with a premature termination codon signal, and indel and splice site variants produce abnormal mRNA due to a frameshift mutation. These abnormal mRNA would be degraded due to the nonsense-mediated decay mechanism or produce a truncated protein. A total of 39 male patients (100%, 39/39) had hemizygous variants in either the CACNA1F or NYX genes. Seventeen patients (100%, 17/17) had a compound heterozygote variant in the *TRPM1*, and three patients (100%, 3/3) had a compound heterozygote variant in the *GRM6* gene (Table 1, Supplemental Table S2).

**Table 1:** Types of novel variations detected in patients with congenital stationary night blindness in this study

Gene	Missense	Nonsense	Frameshift	Deletion	Splicing	Total
*NYX*	3	2	0	2	0	7
*CACNA1F*	4	2	1	1	1	9
*TRPM1*	9	1	0	3	1	14
*GRM6*	2	0	0	0	0	2

### Demographics and clinical characteristics

The majority of our patients were males (91.53%, 54/59). Reduced visual acuity, strabismus and nyctalopia were the main complaints from their parents. BCVA was significantly better in the patients with cCSNB. The median was 20/40 in cCSNB and 20/63 in icCSNB.

**Fig. 2:**
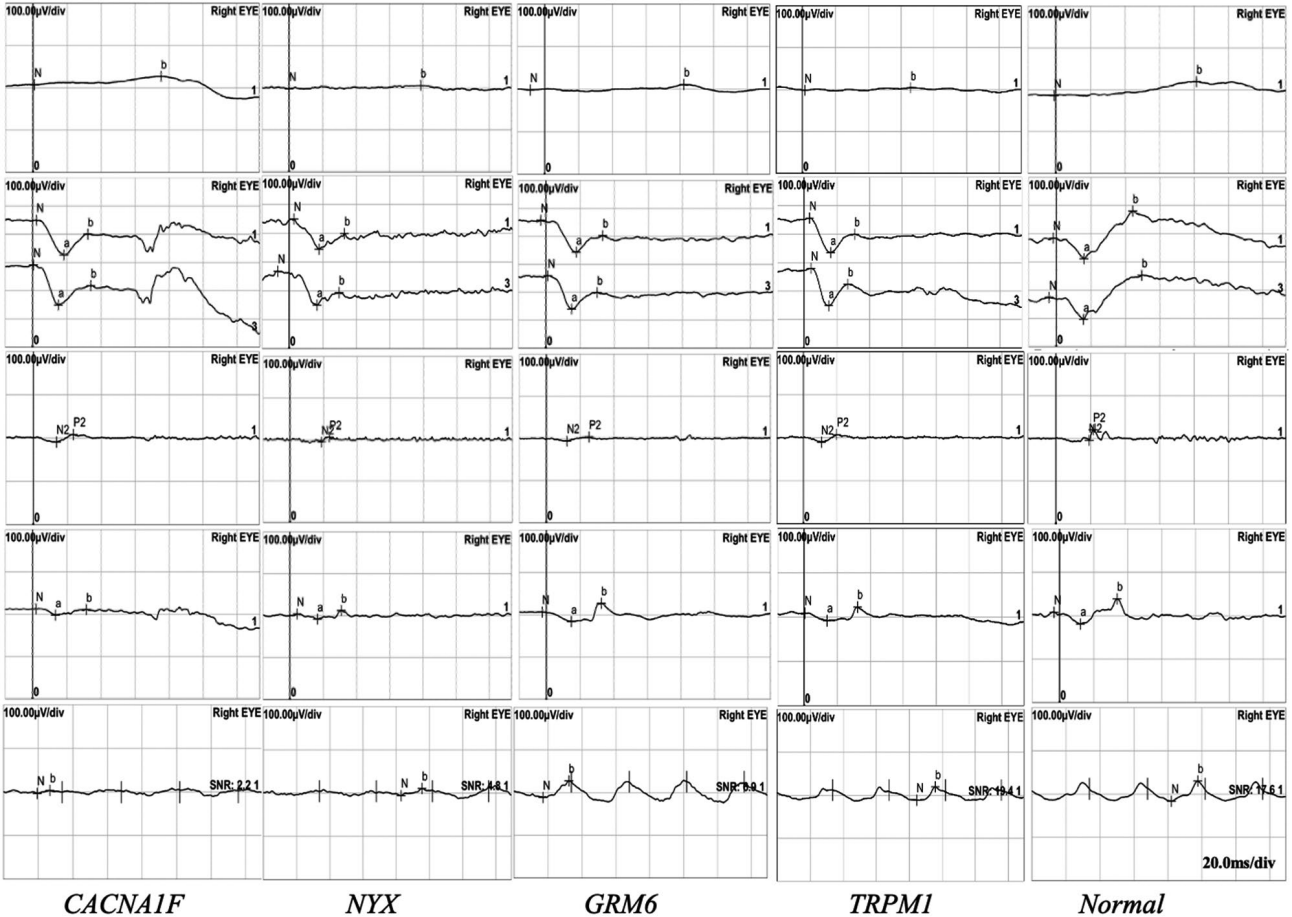
Representative examples of ERG. The ERG trace is recorded in a normal subject and patients with congenital stationary night blindness (CSNB). In dark-adapted and light-adapted conditions, stimulation with a dim flash led to an almost absent rod-specific response in patients with *NYX, GRM6*, and *TRPM1* variations, *CACNA1F* variations. In light-adapted conditions, the response to a standard flash (LA 3.0) had abnormal waveforms with broadened a-waves and biphasic oscillatory potentials in patients with *NYX, GRM6*, and *TRPM1* variations. Patients with *CACNA1F* variation showed substantially reduced a-wave and b-wave amplitude in light-adapted 3.0 and markedly decreased 30-Hz flicker ERG amplitude

Myopia was the most common clinical finding with a high frequency of 96.61% (57/59), followed by nystagmus (62.71%, 37/59) and strabismus (52.54%, 31/59), and nyctalopia (49.15%, 29/59). The incidence rate of high myopia with a SE of less than − 6.0 D reached 66.10% (39/59) at the age of 6 years. Types of strabismus were esotropia (33.90%, 20/59), intermittent exotropia (20.34%, 12/59), and dissociated vertical deviation (DVD) (10.17%, 6/59). A tessellated fundus was more often seen in patients with high myopia of less than − 6.0D. ERG recordings showed different forms of waves based on genotype (Fig. 2).

### Myopia progression

Fifty-seven patients presented with myopia at the age of six years, in which high (< -6 D), moderate (-3 D to -6 D) and mild (0 D to -3 D) myopia accounted for 68.42% (39/57), 24.56% (14/57), and 7.02% (4/57), respectively. The baseline SE at the age of 3 years was − 5.00 ± 3.52 D (range, -13.00 D to 0.00 D), however, the SE increased to -7.51 ± 3.24 D (range, -17.00 D to -2.13 D) at the age of 6 years after a 3-year follow-up. The average of the axial length was 24.58 ± 1.21 mm (range, 22.72 to 28.01 mm) at the age of 3 years, and 25.76 ± 1.00 mm (range, 23.50 to 28.18 mm) at the age of 6 years.

**Fig. 3:**
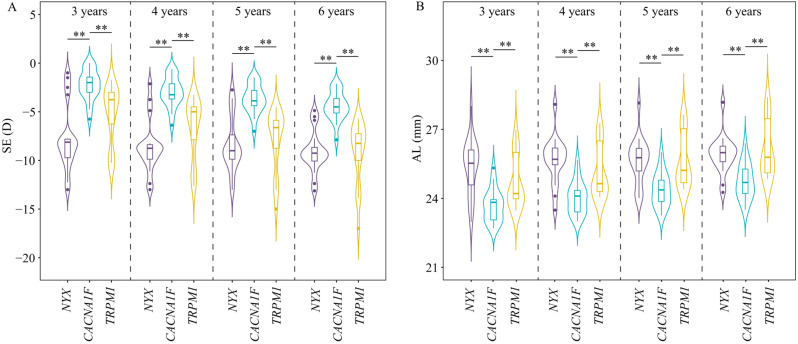
Violin plot with box plot for spherical equivalents (SE) (A) and axial lengths (AL) (B) at age of 3–6 years in CSNB patients with *NYX, CACNA1F*, and *TRPM1* variants. The width of the image reflects the data density, the box plot in the middle represents the median and quartile positions, and the black dots represent outliers. The arrows between the images indicate the nonparametric test results between groups, and “**” indicates *P* < 0.05, which shows statistical significance

At baseline of 3 years old, the mean SE and ocular axis length (AL) were − 7.73 ± 3.37 D and 25.41 ± 1.18 mm, -2.24 ± 1.53 D and 23.68 ± 0.69 mm, and − 5.21 ± 2.89 D and 24.69 ± 1.09 mm in *NYX*, *CACNA1F*, and *TRPM1*, respectively. After 3 years of follow-up, the mean SE and ocular axis were − 9.14 ± 2.09 D and 25.90 ± 0.81 mm, -4.42 ± 1.43 D and 24.74 ± 0.76 mm, and − 9.24 ± 3.16 D and 26.13 ± 1.19 mm in *NYX*, *CACNA1F*, and *TRPM1*, respectively (Table 2). *TRPM1* patients had the highest degree of myopia and longest ocular axis at age of 3 years. By age of 6 years, however, the eyes in the *TRPM1* group had comparable myopia and axial length to those in the *NYX* group. The *CACNA1F* group had relatively lower myopia and axial length than the *NYX* and *TRPM1* groups, both at age 3 and age 6 years (Fig. 3).

**Table 2:** Myopic progression in congenital stationary night blindness patients

Age (years)	NYX	CACNA1F	TRPM1
**Spherical equivalent (D)**			
3	-7.73 ± 3.37	-2.24 ± 1.53	-5.21 ± 2.89
4	-8.27 ± 2.95	-2.96 ± 1.50	-6.68 ± 3.03
5	-8.69 ± 2.50	-3.68 ± 1.44	-7.96 ± 3.15
6	-9.14 ± 2.09	-4.42 ± 1.43	-9.24 ± 3.16
**Axial length (mm)**			
3	25.41 ± 1.18	23.68 ± 0.69	24.69 ± 1.09
4	25.57 ± 1.05	24.01 ± 0.70	25.15 ± 1.11
5	25.73 ± 0.92	24.37 ± 0.72	25.61 ± 1.14
6	25.90 ± 0.81	24.74 ± 0.76	26.13 ± 1.19
**Number of participants**			
	17	20	17

**Fig. 4:**
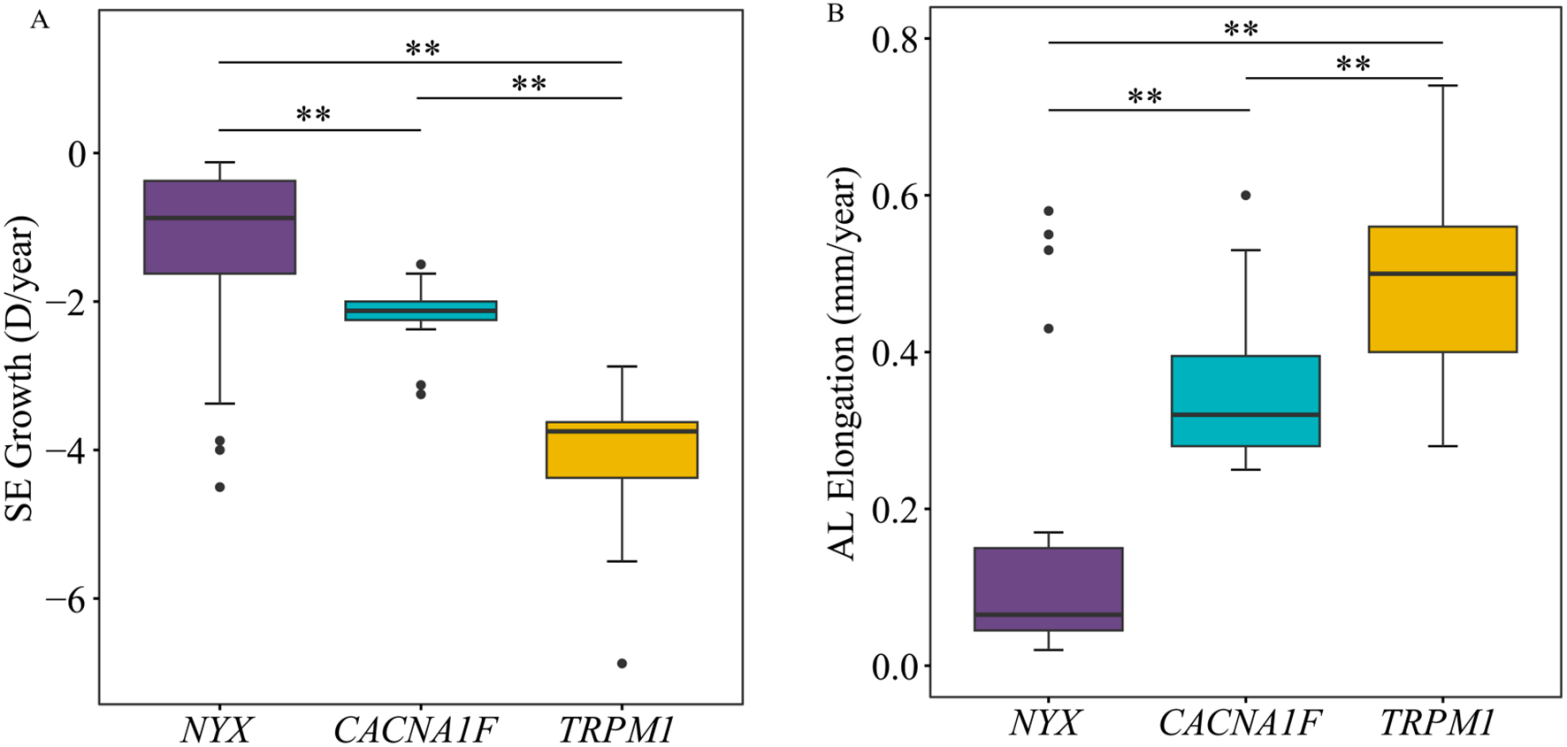
Box plot for spherical equivalents (SE) growth (A) and axial lengths (AL) elongation (B) at a 3-year follow-up in CSNB patients with *NYX, CACNA1F*, and *TRPM1* variants. “**” indicates *P* < 0.05 shows statistical significance using the nonparametric test results between groups

The rates of myopia progression and axial elongation were 0.47 ± 0.48 D/year and 0.16 ± 0.20 mm/year in *NYX*, 0.93 ± 0.24 D/year and 0.35 ± 0.10 mm/year in *CACAN1F*, and 1.35 ± 0.38 D/year and 0.48 ± 0.13 mm/year in *TRPM1*, respectively, in which the rates of myopia progression and axial elongation were the fastest in patients with *TRPM1* variants during 3-year of follow-up (Fig. 4).

## Discussion

In this study, we identified 65 variants from 59 CSNB patients, including 32 novel and 33 previously reported variants. Unlike previous reports on CSNB patients, [6, 11] our patients all were children under 10 years of age. Night blindness was not their principal complaint. Nearly half of the children did not have difficulty in seeing at night. Instead, most of them visited our clinic because of nystagmus, strabismus, and early onset of high myopia. Only two patients with *CACNA1F* variants had hyperopia. However, all our patients underwent genetic testing and were found to have variants in *NYX*, *CACNA1F*, *TRPM1*, and *GRM6* gene. Except for genetic diagnosis, we found that “a triad” of high myopia, nystagmus, and strabismus are prominent clinical features of CSNB and are helpful in diagnosing CSNB.

More than half of the variants (39/65) were detected from the *NYX* and *CACNA1F* genes on the X-chromosome, followed by variants in *TRMP1* on an autosome, and few variants were found in *GRM6*. These genes are mainly expressed in the photoreceptors, bipolar and amacrine interneurons, and ganglion cells in the retina, and play essential roles in the transmission of signaling cascade from the photoreceptors (PCs) to the bipolar cells (BCs) [12]. Patients with cCSNB have no residual rod function, whereas patients with icCSNB show reduced but recordable both cone and rod function. Some icCSNB patients reportedly had a double peak in the 30 Hz flicker ERG [11]. However, we did not find this “double peak” pattern in the 30-Hz flicker ERG of patients with icCSNB.

Transmission of light-induced cascade signals via the PC-BC connection is an important link in the formation of human vision, which relies on the release of the neurotransmitter glutamate into synapses between the PC and BC, where the rod cell synapses predominantly with ON bipolar cells and the cone cell synapses with both ON- and OFF-bipolar cells. The glutamate released from the PC binds to the mGluR6 receptor encoded by the *GRM6* gene on the dendrites of bipolar cells and initiates depolarizing response generation in ON bipolar cells. As a component of the mGluR6 signaling pathway, TRPM1 modulates the susceptibility of ON-bipolar cells to depolarizing responses by opening and closing the TRPM1 channel to mediate the influx of calcium ions into bipolar cells [13]. Meanwhile, the encoded protein of Nyctalopin by the *NYX* gene is responsible for the correct localization of the TRPM1 channel to the synapse at dendritic tips [14]. 

Mutations of the *NYX*, *GRM6*, and *TRPM1* will result in dysfunction of the ON-bipolar cell, leading to the cCSNB with a reduced b-wave. Unlike the aforementioned genes, *CACNA1F* encodes a voltage-dependent L-type calcium channel subunit alpha-1 F, which is expressed in both the rod and cone active regions and plays an important role in mediating the influx of calcium ions into photoreceptor cells [15]. Mutations in this gene can cause dysfunction in both cone and rod cells due to the ion channels located within the presynaptic membrane of the photoreceptors, and affect both ON and OFF pathways where the cone synapses directly with both types of bipolar cells, leading to icCSNB. This contrasts with complete CSNB, where dysfunction selectively affects ON bipolar cells. In dark-adapted conditions, stimulation with a dim flash led to an almost absent rod-specific response in patients with *NYX, GRM6*, and *TRPM1* variations, while low-amplitude response in patients with *CACNA1F* variations. In light-adapted conditions, the response to a standard flash (light-adapted 3.0) had abnormal waveforms with broadened a-waves and biphasic oscillatory potentials, but preserved light-adapted 3.0 and 30-Hz flicker ERG b-wave amplitude in patients with *NYX*, *GRM6*, and *TRPM1* variations. However, patients with *CACNA1F* variation showed substantially reduced a-wave and b-wave amplitude in light-adapted 3.0 and markedly decreased 30-Hz flicker ERG amplitude, which demonstrated both severe cone and rod dysfunction.

Dysfunctions in ON-BC signaling have also been suggested to be associated with the development of refractive errors [16]. Many patients with cCSNB are found to have myopia, especially in those patients carrying with GRM6 [17] and *NYX* mutations [18]. In addition, even without night blindness, some patients with *NYX* mutations may have myopia [19, 20]. However, the molecular basis of the ON-BC signaling cascade associated with myopia is not clear and may be caused by changes in several key factors, including the levels of the neurotransmitter dopamine and the molecules that connect mGluR6 signaling to the pathway that controls axial growth [21]. 

A previous study showed that the change in refractive error varied between − 2.2 D and + 1.3 D every 5 years, with one outlier of -3.6 D in 5 years [11]. However, we found that myopia developed very quickly in those patients with CSNB, varied from a baseline of -4.98 ± 3.41D to -7.81 ± 1.24 D after a 3-year follow-up, with elongation of the ocular axis from 24.64 ± 1.18 mm to 25.63 ± 1.06 mm. A previous report showed that the mean SE change was 0.81 ± 0.53 D/year with a mean increase in AL of 0.41 ± 0.22 mm/year in children aged 4 to 12 years [22]. In our patients, the mean SE change was 0.47 ± 0.48 D/year and a mean increase in AL was 0.16 ± 0.20 mm/year in NYX, 0.93 ± 0.24 D/year and 0.35 ± 0.10 mm/year in CACAN1F, and 1.35 ± 0.38 D/year and 0.48 ± 0.13 mm/year in TRPM1, respectively. The associated myopia was mainly axial. Early-onset myopia was more common in patients with mutations of *NYX, GRM6*, and *TRPM1* than in patients with *CACNA1F* mutations, further supporting the link between the mGluR6 signaling pathway and myopia [23]. Animal experiments also supported that blockade of ON-BC transmission [24] or genetic elimination of NYX or mGluR6 would exacerbate the development of refractive error [25, 26]. 

The study did not categorize whether the children had received any treatment or specific means of myopia control during the 3-year period, which represents a major limitation.

In summary, we described the clinical characteristics of a cohort of patients with CSNB, investigated the pathogenic variants in the genes associated with CSNB, and evaluate the progression of myopia in patients with different genetic backgrounds in this study. We found that myopia and strabismus are prominent clinical features that can be helpful in the diagnosis of CSNB. In addition, we identified 45 novel pathogenic variants in the genes *NYX*, *CACNA1F*, *TRPM1*, and *GRM6*. High myopia was more frequent in patients with *NYX*, but the rate of progression was not greater in patients with *NYX* than in those with *TRPM1*. The possible pathophysiology related to different myopic progression in CSNB patients may be due to the background of different gene variants.

### Electronic supplementary material

Below is the link to the electronic supplementary material.

**Supplementary Material 1**: Links of fastq data

**Supplementary Material 2**: Pedigrees of the families with variants in CSNB genes. Filled symbols indicate individuals affected with CSNB.

**Supplementary Material 3**: Chromatograms showing novel variants identified in CSNB patients.

**Supplementary Material 4**: Table S2.CSNB-related variations detected in this study.

**Supplementary Material 5**: 3-D structure of protein using the PyMOL program.

**Supplementary Material 6**: Table S1. Novel variations detected in patients with congenital stationary night blindness in this study.

## Data Availability

The original contributions presented in the study are included in the Supplementary Material, part in https://www.biosino.org/download/node/data/public/OED883951, further inquiries can be directed to the corresponding author.
