# Supplementary material for: Clinical and genetic studies for a cohort of patients with congenital stationary night blindness
Source: Orphanet J Rare Dis. 2024 Mar 6;19:101. doi: 10.1186/s13023-024-03091-3 (PMC10918914; doi:10.1186/s13023-024-03091-3)
Supplement: Supplementary file 2 — Supplementary Material 2: Pedigrees of the families with variants in CSNB genes. Filled symbols indicate individuals affected with CSNB. [file 13023_2024_3091_MOESM2_ESM.docx]

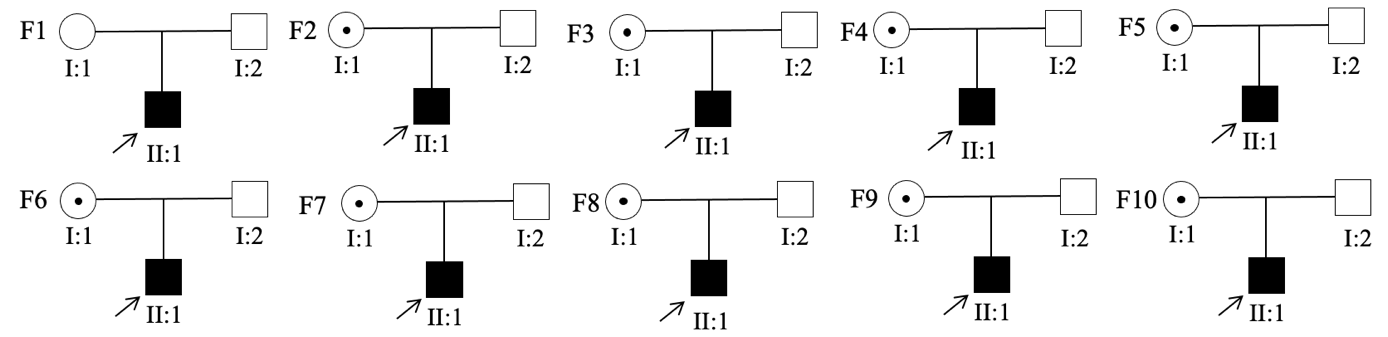

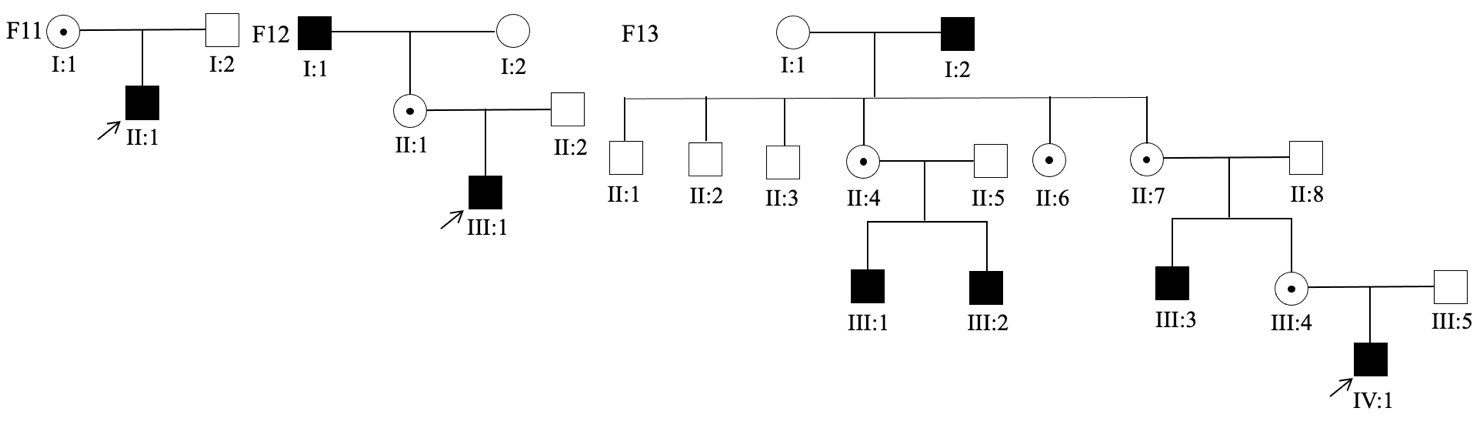

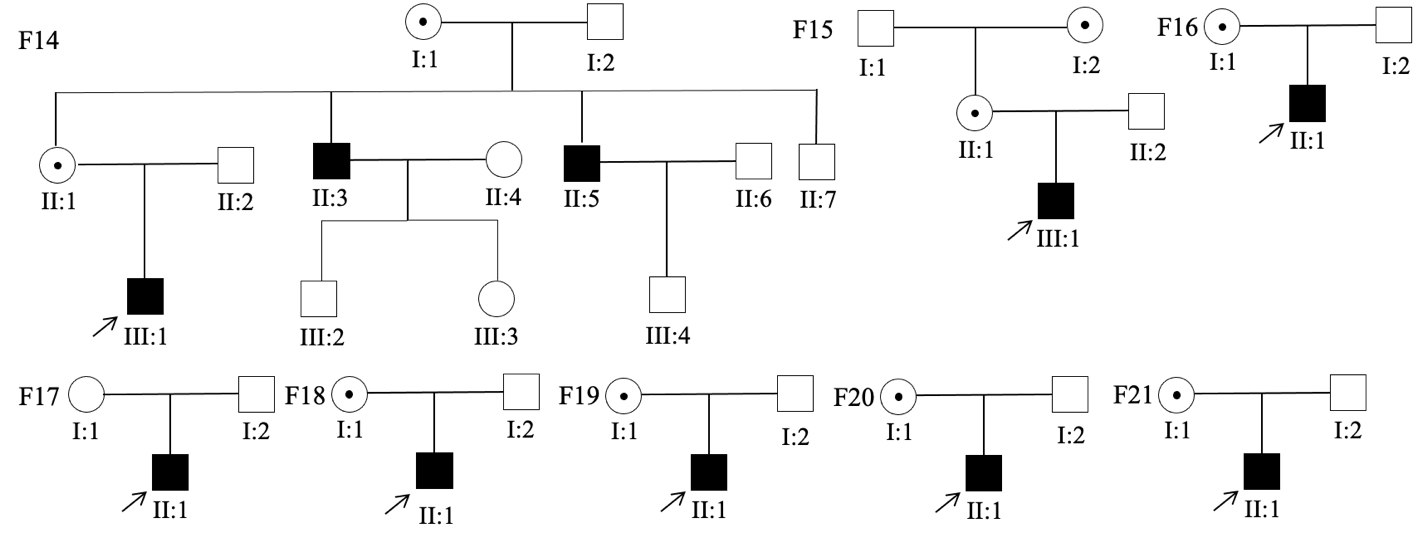

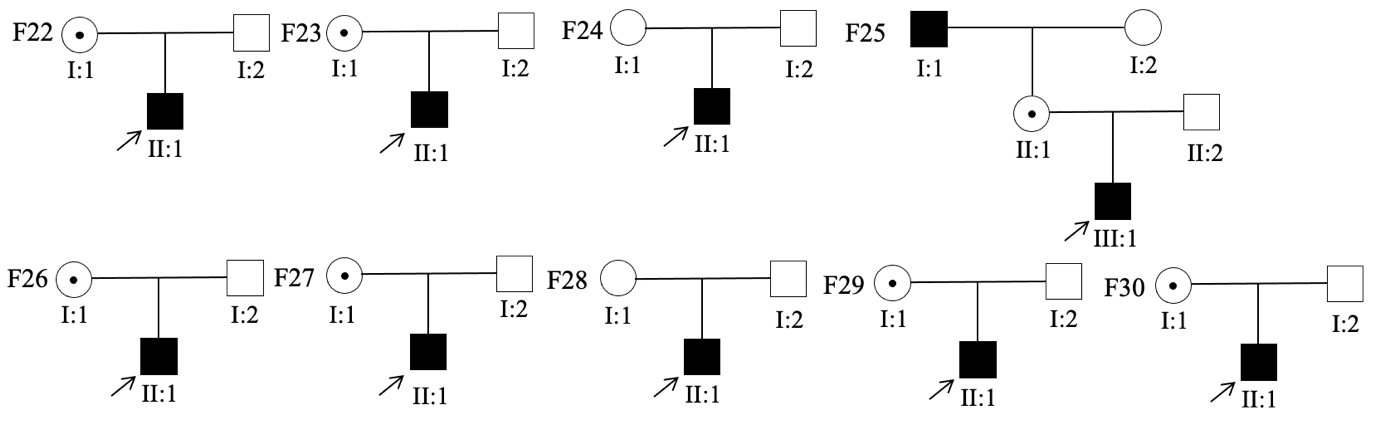

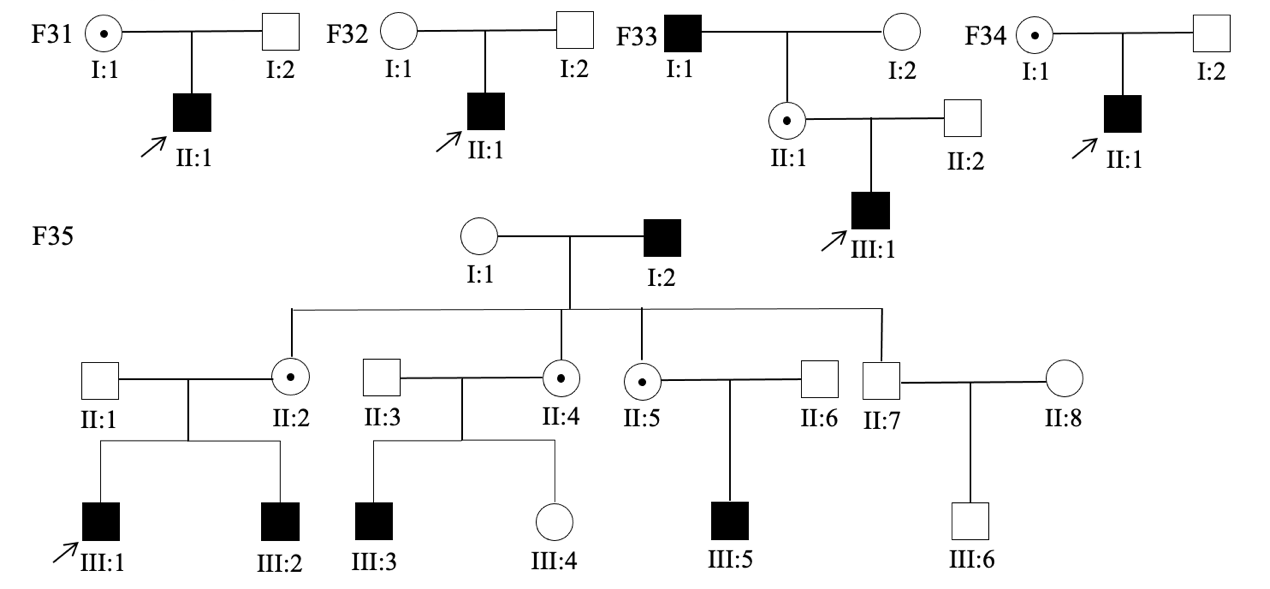

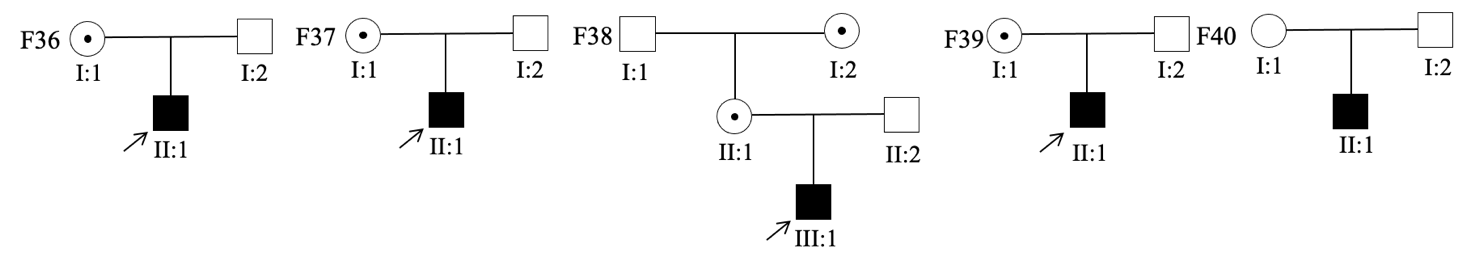

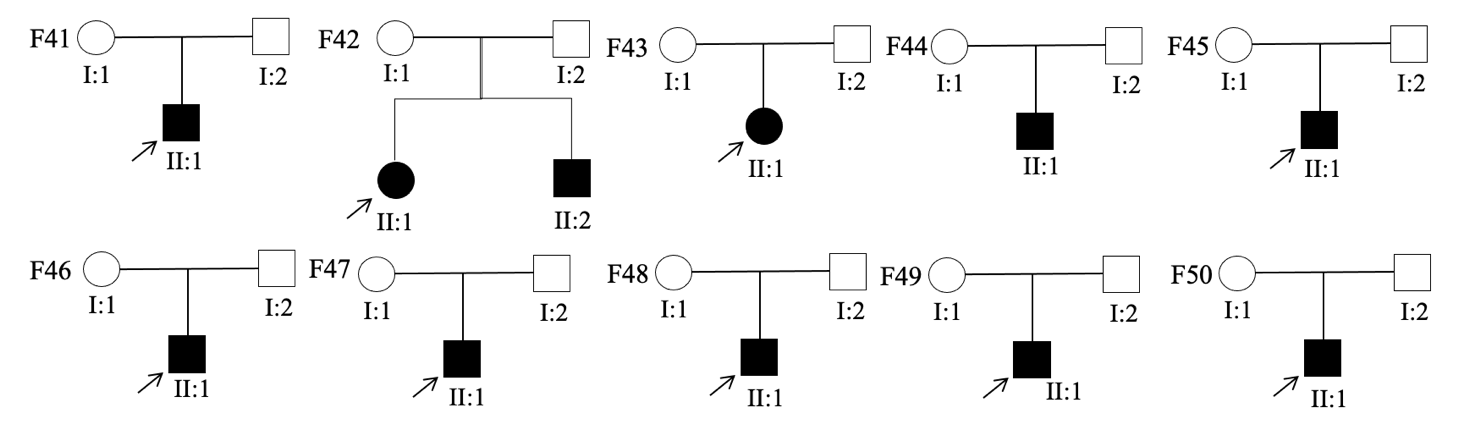

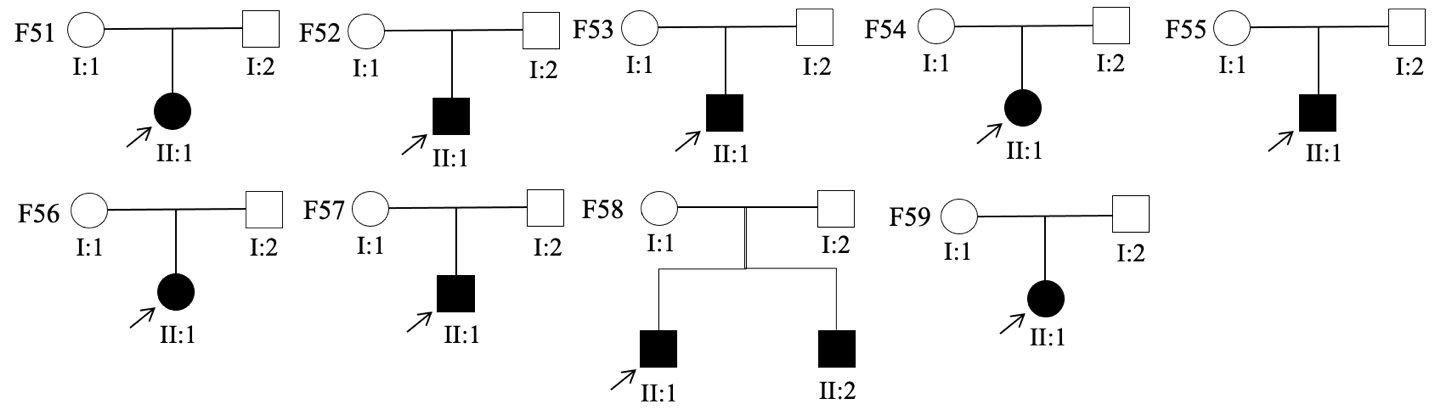


**Supplement Figure** Pedigrees of the families with variants in CSNB genes. Filled symbols indicate individuals affected with CSNB.
