# Supplementary material for: Clinical and genetic studies for a cohort of patients with congenital stationary night blindness
Source: Orphanet J Rare Dis. 2024 Mar 6;19:101. doi: 10.1186/s13023-024-03091-3 (PMC10918914; doi:10.1186/s13023-024-03091-3)
Supplement: Supplementary file 3 — Supplementary Material 3: Chromatograms showing novel variants identified in CSNB patients. [file 13023_2024_3091_MOESM3_ESM.docx]

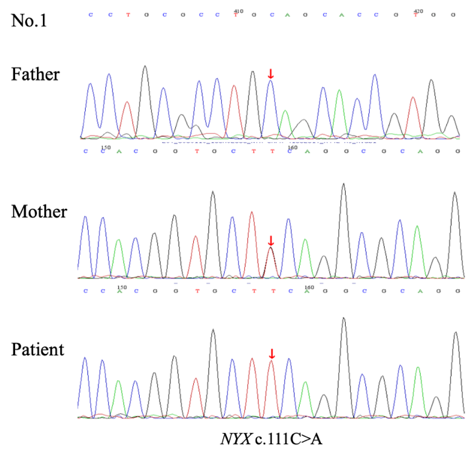

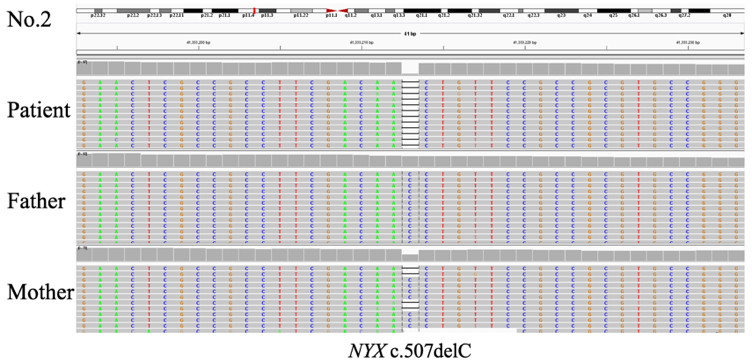

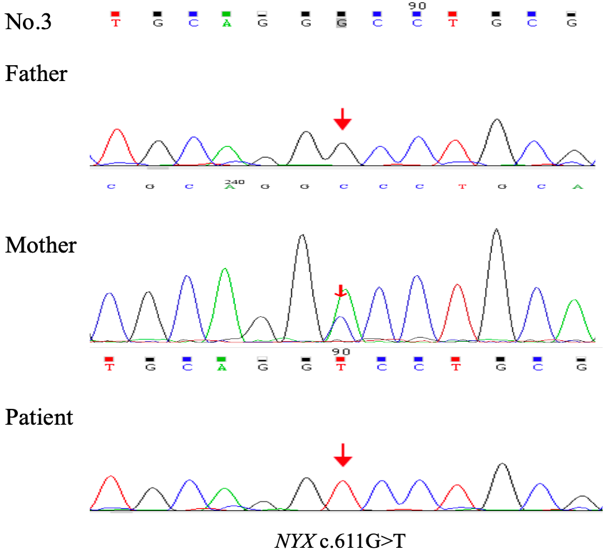

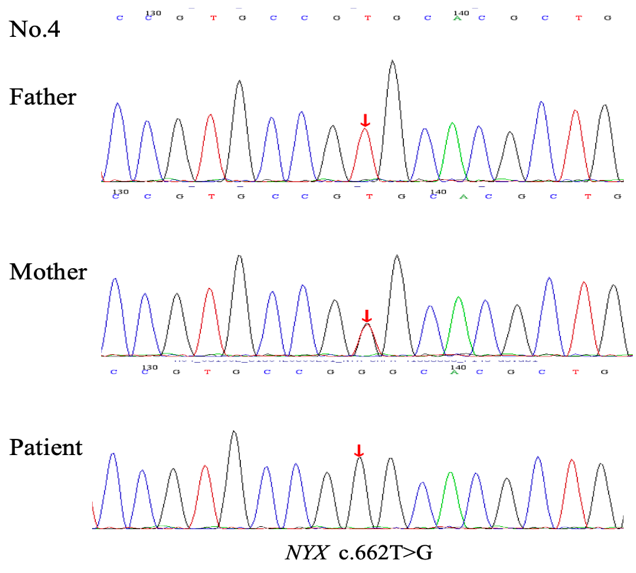

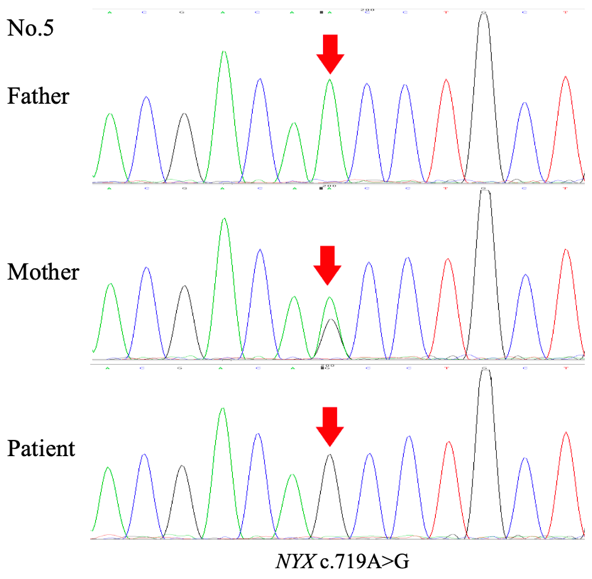

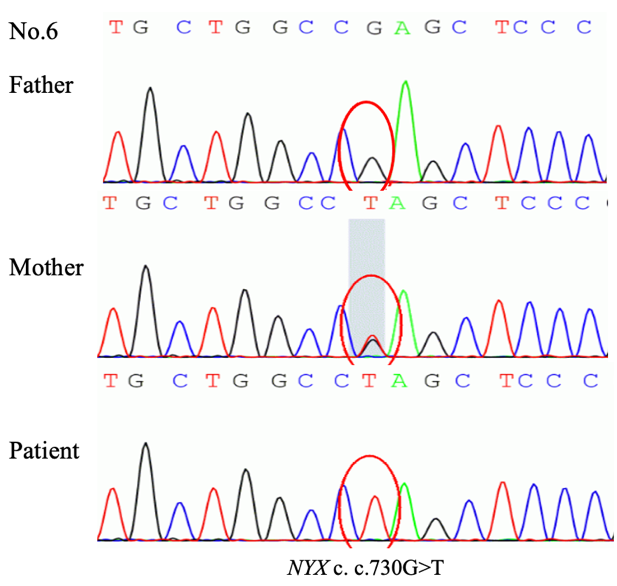

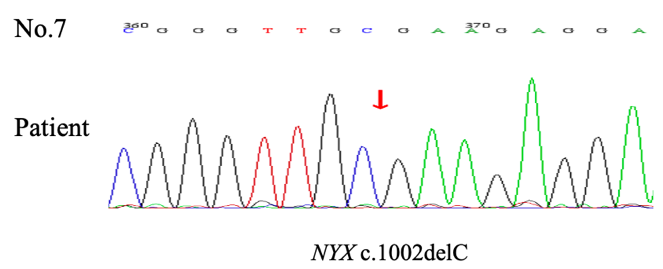

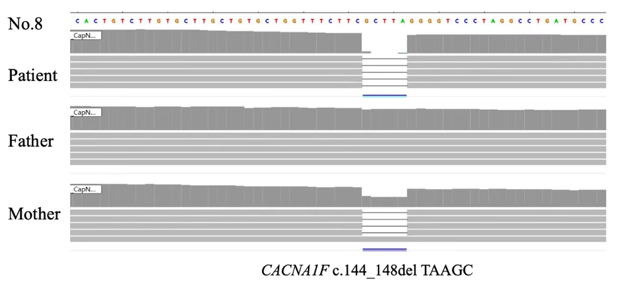

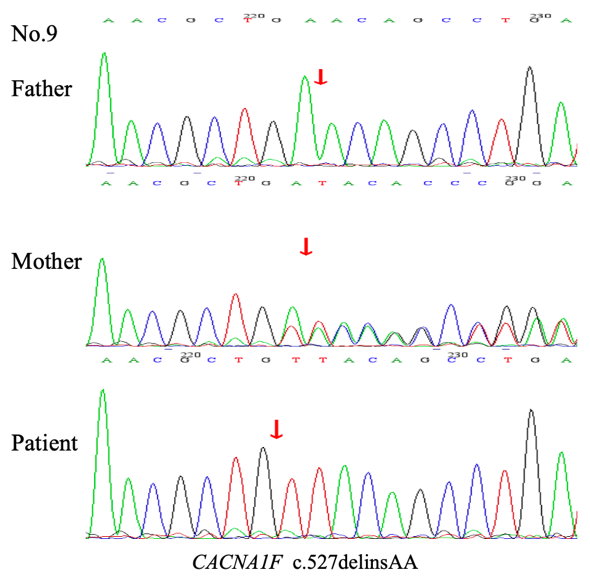

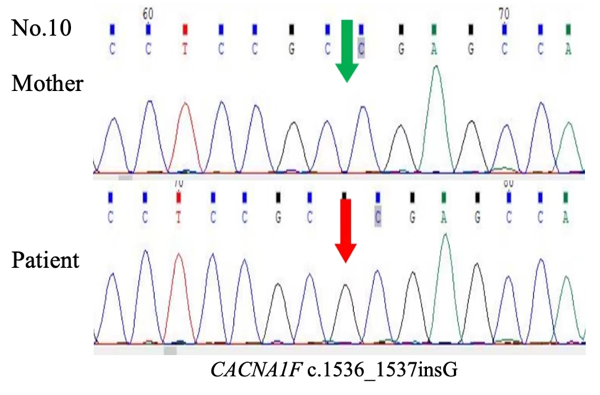

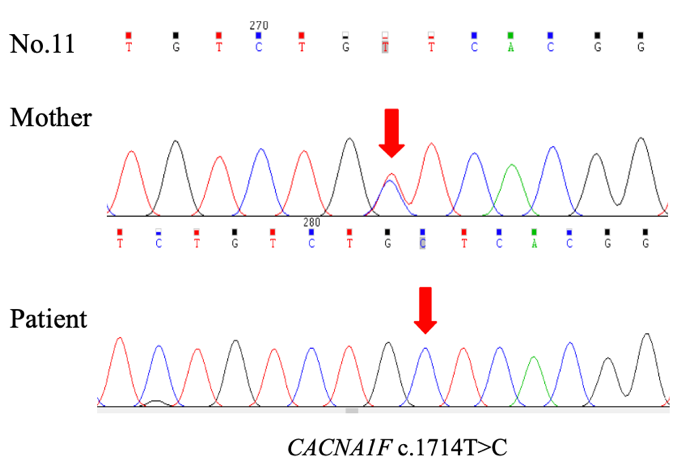

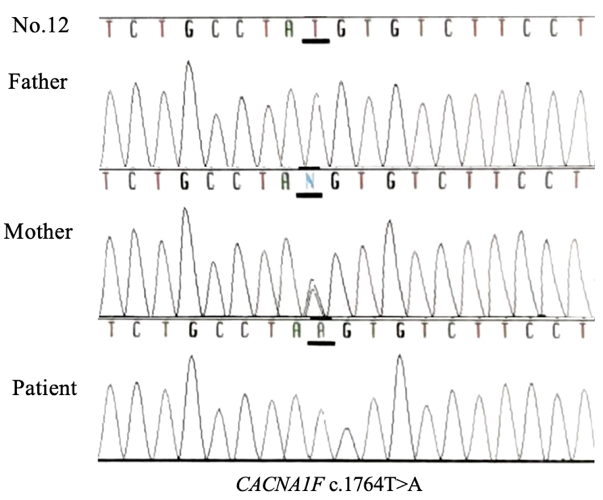

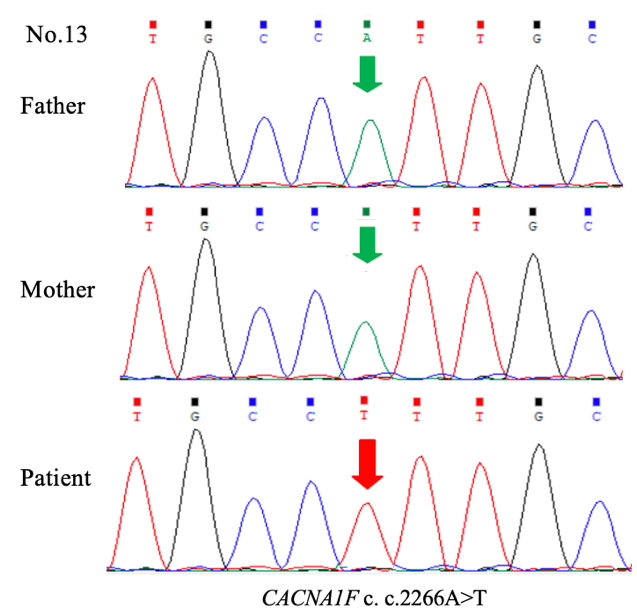

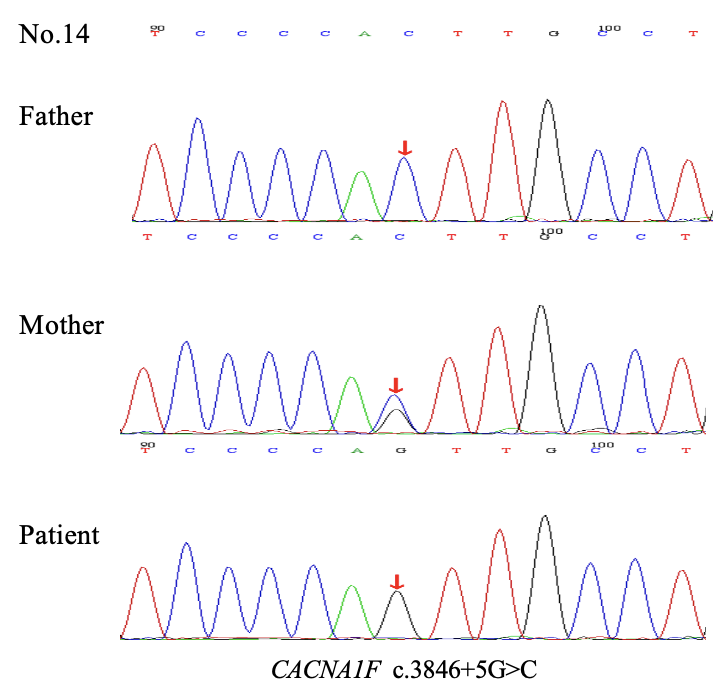

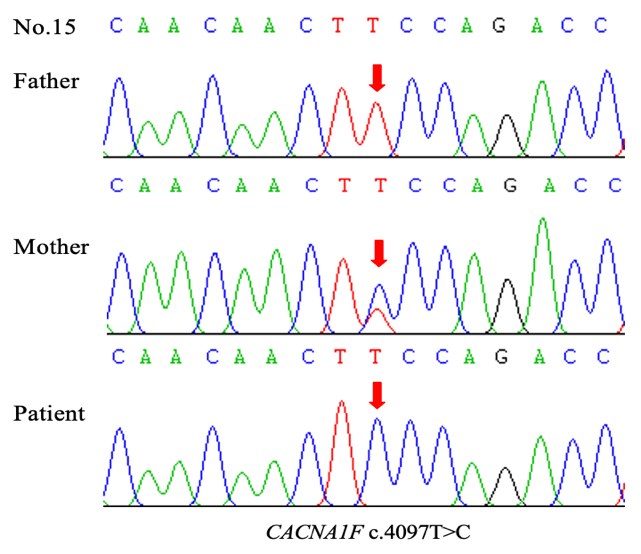

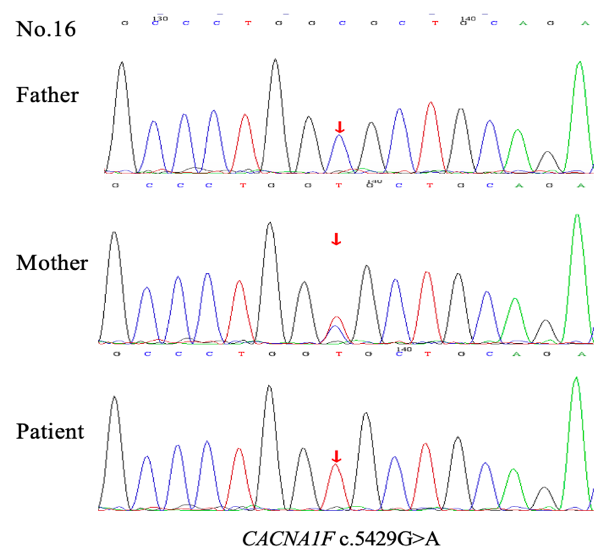

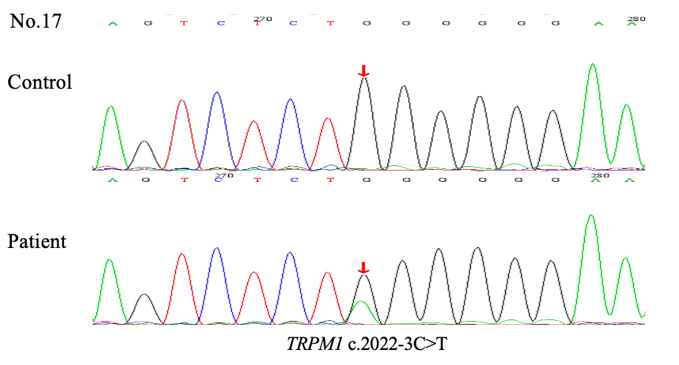

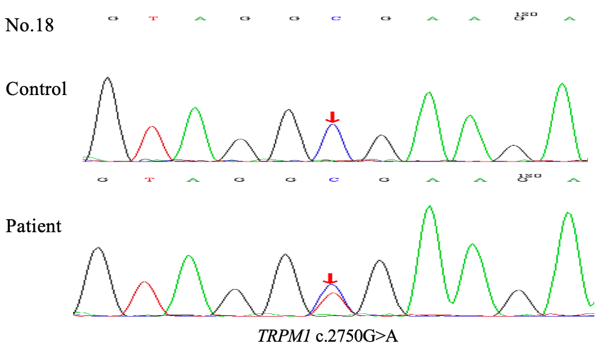


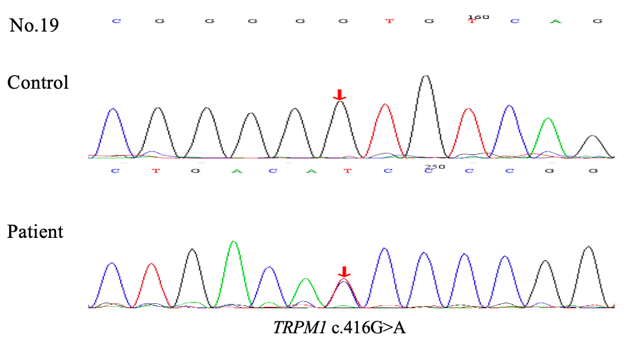

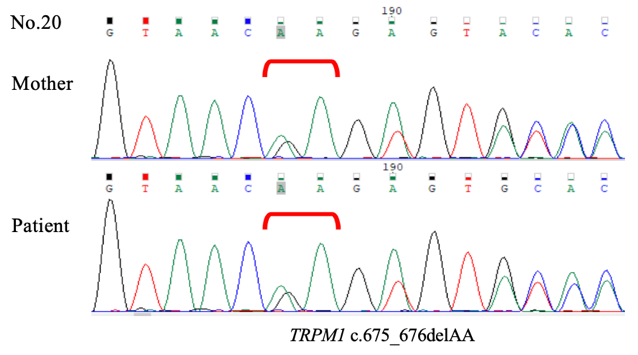

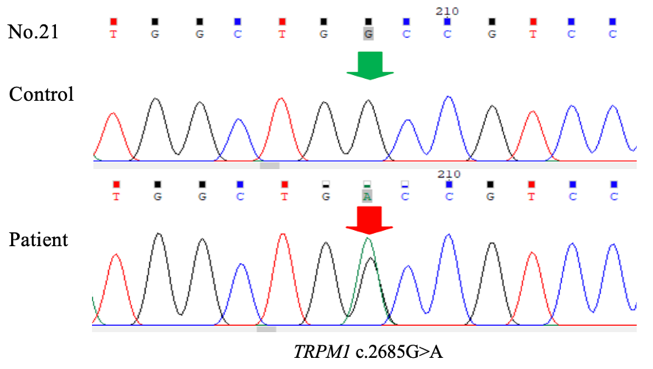

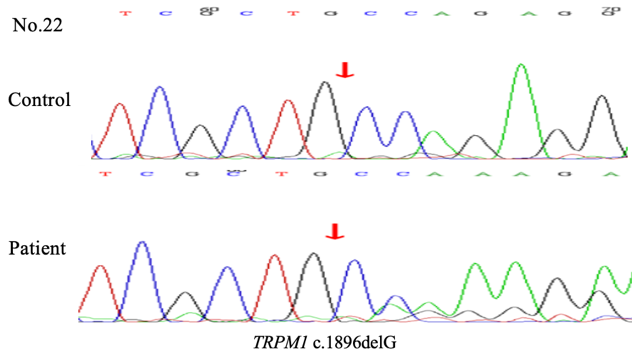

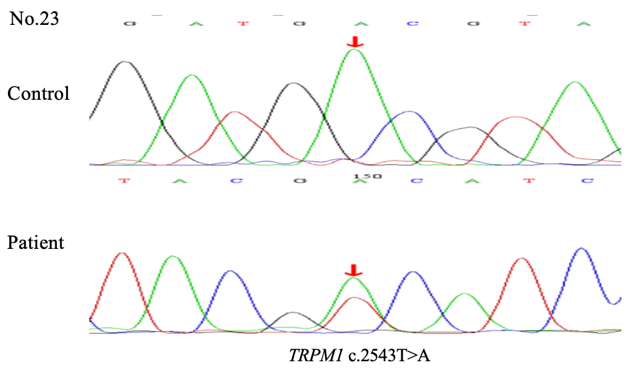

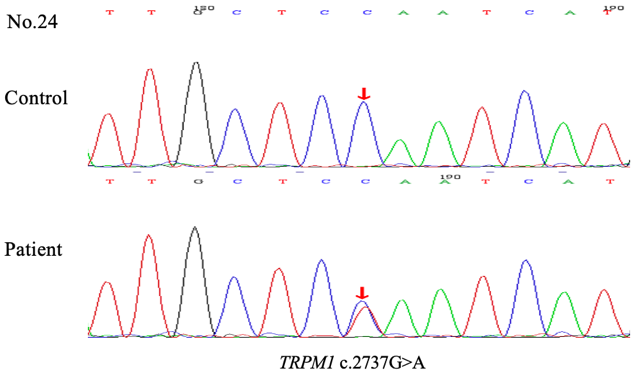

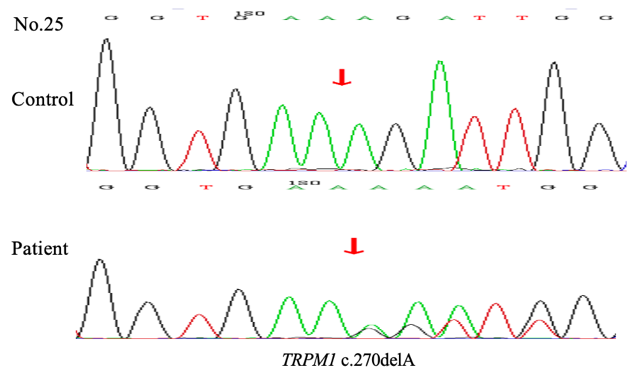

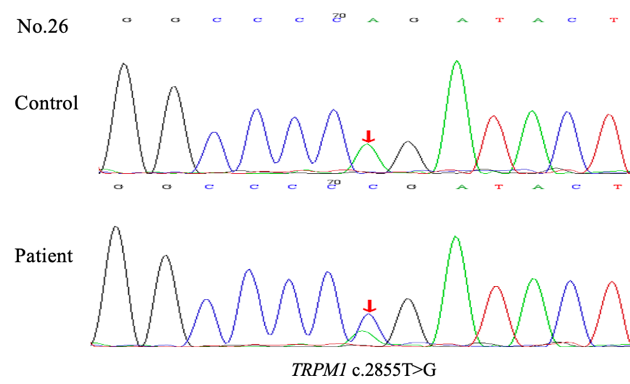

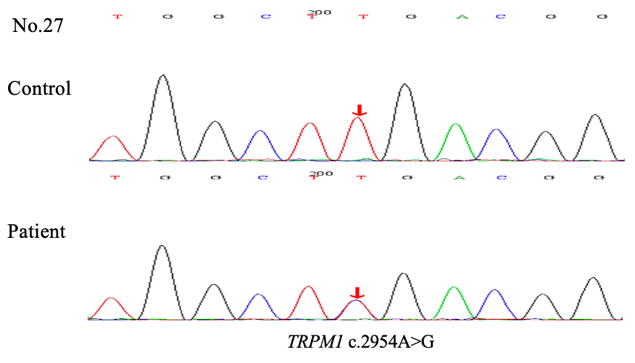

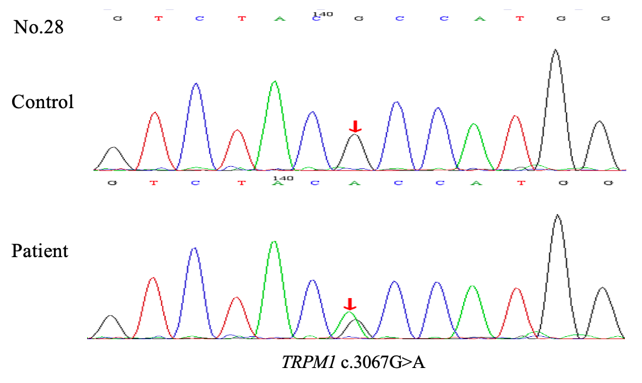

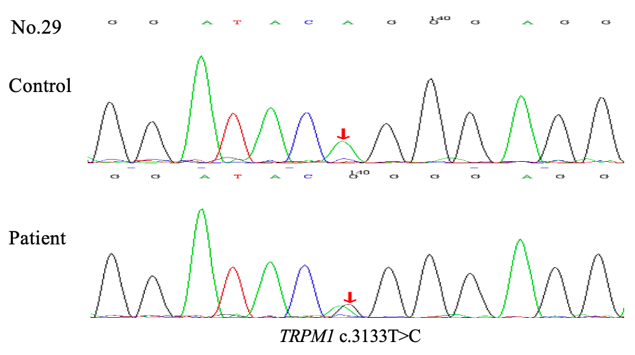

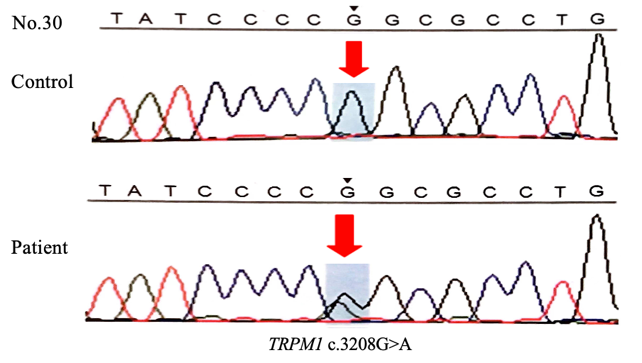

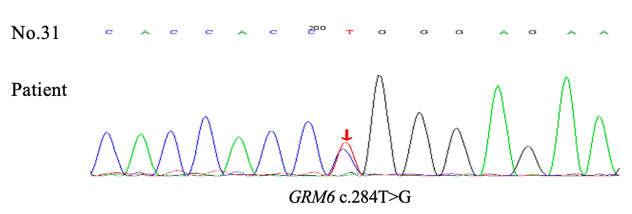

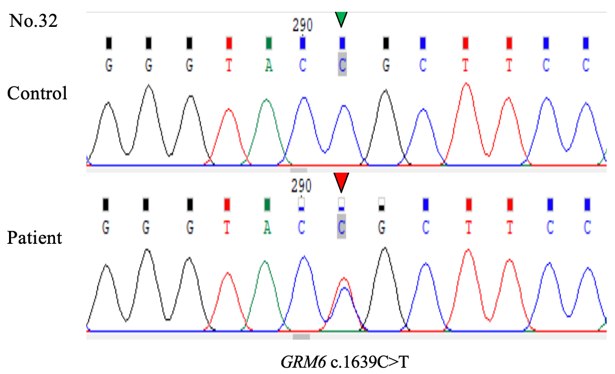


**Supplement Figure** Chromatograms showing novel variants identified in CSNB patients. No.2 and No.8 display the variation by observation of the bam file using Integrative Genomics Viewer (IGV) software.
