# Supplementary material for: Clinical and genetic studies for a cohort of patients with congenital stationary night blindness
Source: Orphanet J Rare Dis. 2024 Mar 6;19:101. doi: 10.1186/s13023-024-03091-3 (PMC10918914; doi:10.1186/s13023-024-03091-3)
Supplement: Supplementary file 4 — Supplementary Material 4: Table S2.CSNB-related variations detected in this study. [file 13023_2024_3091_MOESM4_ESM.docx]

Table S2 CSNB-related variations detected in this study

| **No.** | **Gender** | **Gene** | **Location** | **Nucleotide** | **Protein** | **Source** | **Type** | **SIFT** | **Polyphen-2** | **CADD** | **ACMG** | **Evidence levels** | SpliceAI | **Report** |
| --- | --- | --- | --- | --- | --- | --- | --- | --- | --- | --- | --- | --- | --- | --- |
| 1 | M | *NYX* | Exon2 | c.85_108del | p.R29_A36del | Mother | Deletion | - | - | - | Pathogenic | PVS1+PM2+PP4 | - | PMID: 12552565 |
| 2 | M | *NYX* | Exon2 | c.111C>A | p.C37* | Mother | Nonsense | - | - | - | Pathogenic | PVS1+PM2+PP4 | - | Novel |
| 3 | M | *NYX* | Exon2 | c.143G>A | p.Cys48Tyr | Mother | Missense | D | P | 25.8 | VUS | PS4+PM2+PP3+PP4 | - | PMID:  19578023 |
| 4 | M | *NYX* | Exon2 | c.149G>C | p.Arg50Pro | Mother | Missense | D | P | 26.3 | VUS | PS4+PM2+PP3+PP4 | - | PMID:  22735794 |
| 5 | M | *NYX* | Exon2 | c.214A>C | p.Asn72His | Mother | Missense | D | D | 24.3 | Likely pathogenic | PP1+PM2+PP3+ PS4+PP4 | - | PMID:  26234941 |
| 6 | M | *NYX* | Exon2 | c.281G>C | p.Arg94Pro | Mother | Missense | T | P | 23.7 | Likely pathogenic | PP1+PM2+PP4 | - | PMID:  16670814 |
| 7 | M | *NYX* | Exon2 | c.283delC | p.His95Thrfs*46 | Mother | Deletion | - | - | - | Pathogenic | PVS1+PP1+PM2+PP4 | - | PMID:  31826698 |
| 8 | M | *NYX* | Exon2 | c.368T>G | p.L123R | Mother | Missense | D | P | 24.7 | Likely pathogenic | PM2+PP3+PS4+PP4 | - | PMID:  25307992 |
| 9 | M | *NYX* | Exon2 | c.371_377delGCTACCT | Y125Tfs*14 | Mother | Deletion | - | - | - | Pathogenic | PVS1+PP1+PM2+PP4+ PS4 | - | PMID:  26234941 |
| 10 | M | *NYX* | Exon2 | c.507delC | p.L170Cfs*15 | Mother | Deletion | - | - | - | Pathogenic | PVS1+PM2+PP4 | - | Novel |
| 11 | M | *NYX* | Exon2 | c.518G>C | p.R173P | Mother | Missense | T | D | 24.3 | Likely pathogenic | PM2+PP3+PP1+ PS4+PP4 | - | PMID: 19578023 |
| 12 | M | *NYX* | Exon2 | c.553A>C | p.T185P | Mother | Missense | T | D | 23.6 | Likely pathogenic | PM2+PP3+PP4 | - | Novel |
| 13 | M | *NYX* | Exon2 | c.611G>T | p.G204V | Mother | Missense | D | D | 25.3 | Likely pathogenic | PM2+PP3+PP4 | - | Novel |
| 14 | M | *NYX* | Exon2 | c.662T>G | p.V221G | Mother | Missense | D | D | 23.6 | Likely pathogenic | PM2+PP3+PP4 | - | Novel |
| 15 | M | *NYX* | Exon2 | c.719A>G | p.N240S | Mother | Missense | D | D | 23.8 | Likely pathogenic | PM2+PP3+PP4 | - | Novel |
| 16 | M | *NYX* | Exon2 | c.730G>T | p.E244* | Mother | Nonsense | - | - | - | Pathogenic | PVS1+PM2+PP4 | - | Novel |
| 17 | M | *NYX* | Exon2 | c.1002delC | p.R335Afs*13 | Unknown | Deletion | - | - | - | Pathogenic | PVS1+PM2+PP4 | - | Novel |
| 18 | M | *CACNA1F* | Exon2 | c.144_148delTAAGC | p.R50Kfs*66 | Mother | Deletion | - | - | - | Likely pathogenic | PVS1+PM2 | - | Novel |
| 19 | M | *CACNA1F* | Exon2 | c.245G>A | p.R82Q | Mother | Missense | D | D | 31 | Likely pathogenic | PS4+PM2+PP3+PP4 | - | PMID:  28002560 |
| 20 | M | *CACNA1F* | Exon4 | c.448G>C | p.G150R | Mother | Missense | D | D | 33 | Likely pathogenic | PS4+PM2+PP3+PP4 | - | PMID:  12187427 |
| 21 | M | *CACNA1F* | Exon4 | c.527delinsAA | p.F176* | Mother | Nonsense | - | - | - | Pathogenic | PVS1+PM2+PP4 | - | Novel |
| 22 | M | *CACNA1F* | Exon7 | c.952_954delTTC | p.F318del | Mother | Deletion | - | - | - | Likely pathogenic | PS4+PM2+PM4 | - | PMID:  11281458 |
| 23 | M | *CACNA1F* | Exon10 | c.1282C>T | p.Gln428* | Mother | Nonsense | - | - | - | Pathogenic | PVS1+PS4+PM2+PP4 | - | PMID:  12111638 |
| 24 | M | *CACNA1F* | Exon13 | c.1536_1537insG | p.R513Afs*37 | De novo | Insertion | - | - | - | Pathogenic | PVS1+PS2+PM2 | - | Novel |
| 25 | M | *CACNA1F* | Exon14 | c.1714T>C | p.F572L | Mother | Missense | D | D | 26.7 | VUS | PM2+PP3+PP4 | - | Novel |
| 26 | M | *CACNA1F* | Exon14 | c.1764T>A | p.Tyr588* | Mother | Nonsense | - | - | - | Pathogenic | PVS1+PM2+PP4 | - | Novel |
| 27 | M | *CACNA1F* | Exon15 | c.2012C>G | p.S671C | Mother | Missense | D | P | 25.3 | VUS | PM2+PP3 | - | PMID: 33691693 |
| 28 | M | *CACNA1F* | Exon17 | c.2266A>T | p.I756F | De novo | Missense | D | D | 25.8 | VUS | PM2+PP3 | - | Novel |
| 29 | M | CACNA1F | Exon24 | c.2905C>T | p.R969X | Mother | Nonsense | - | - | - | Pathogenic | PVS1+PS4+PM2+PP4 | - | PMID:  9662399 |
| 30 | M | *CACNA1F* | Exon24 | c.2932C>T | p.R978* | Mother | Nonsense | - | - | - | Pathogenic | PVS1+PS4+PM2+PP4 | - | PMID:  23714322 |
| 31 | M | *CACNA1F* | Exon26 | c.3089C>T | p.T1030M | Mother | Missense | D | D | 31 | VUS | PS4+PM2+PP3+PP4 | - | PMID: 33994118 |
| 32 | M | *CACNA1F* | Exon27 | c.3178C>T | p.R1060W | Mother | Missense | D | D | 33 | Likely pathogenic | PS4+PS3+PM2+PP3+PP4 | - | PMID:  9662399 |
| 33 | M | *CACNA1F* | Exon31 | c.3758C>T | p.A1253V | Mother | Missense | T | P | 23.1 | VUS | PM2+PP4 | - | PMID: 25307992 |
| 34 | M | *CACNA1F* | Intron32 | c.3846+5G>C |  | Mother | Splicing | - | - | - | VUS | PM2+PP3+PP4 | AG (0.56) | Novel |
| 35 | M | *CACNA1F* | Exon35 | c.4097T>C | p.F1366S | Mother | Missense | D | D | 27 | Likely pathogenic | PM2+PP3+PP4 | - | Novel |
| 36 | M | *CACNA1F* | Exon39 | c.4594C>T | p.R1532W | Mother | Missense | D | D | 27.4 | Likely pathogenic | PS4+PM2+PP3 |  |  |
| 37 | M | *CACNA1F* | Exon42 | c.4916_4917delCA | p.T1639Rfs*16 | Mother | Missense | D | P | 27.8 | VUS | PS4+PM2+PP3+PP4 | - | Novel |
| 38 | M | *CACNA1F* | Exon46 | c.5429G>A | p.R1810H | Mother | Missense | D | B | 34 | VUS | PM2+PP4 | - | Novel |
| 39 | M | *CACNA1F* | Exon48 | c.5594G>A | p.Arg1865His | Mother | Missense | T | B | 19.9 | LB | BS2+BP4 | - | PMID: 11381068 |
| 40 | M | *TRPM1* | Exon4 | c.215A>G | p.Y72C | Father | Missense | D | P | 26.1 | Likely pathogenic | PM3+PM2+PP3+PP4 | - | PMID:  19896113 |
|  |  | *TRPM1* | Intron16 | c.2022-3C>T |  | Mother | Splicing | - | - | - | VUS | PM2 | AG (0.21) | Novel |
| 41 | M | *TRPM1* | Exon4 | c.215A>G | p.Y72C | Father | Missense | D | P | 26.1 | Likely pathogenic | PM3+PM2+PP3+PP4 | - | PMID:  19896113 |
|  |  | *TRPM1* | Exon21 | c.2750G>A | p.R917H | Mother | Missense | D | D | 34 | VUS | PM3+PM2+PP3+PP4 | - | Novel |
| 42 | F | *TRPM1* | Exon4 | c.416G>A | p.G139D | Mother | Missense | D | D | 28.4 | VUS | PM2+PP3 | - | Novel |
|  |  | *TRPM1* | Intron2 | c.18-3C>G |  | Father | Splicing | - | - | - | Likely pathogenic | PS3+PM2+PP4 | AL (0.83) | PMID: 20300565 |
| 43 | F | *TRPM1* | Exon6 | c.675_676del | p.R226Sfs*11 | Mother | Deletion | - | - | - | Pathogenic | PVS1+PM2+PP4 | - | Novel |
|  |  | *TRPM1* | Exon20 | c.2685G>A | p.Trp895* | Father | Nonsense | D | D | 33.4 | VUS | PM3+PM2+PP3+PP4 | - | Novel |
| 44 | M | *TRPM1* | Exon16 | c.1870C>T | p.R624C | Mother | Missense | T | D | 31.1 | VUS | PM2+PP3+PP4 | - | PMID:  20300565 |
|  |  | *TRPM1* | Exon4 | c.296T>C | p.L99P | Father | Missense | D | P | 25.3 | Likely pathogenic | PM3+PM2+PP3+PP4 | - | PMID:  29522070 |
| 45 | M | *TRPM1* | Exon16 | c.1896delG | p.W632fs*53 | Father | Deletion | - | - | - | Pathogenic | PVS1+ PM2+PP4 | - | Novel |
|  |  | *TRPM1* | Exon20 | c.2543T>A | p.V848D | Mother | Missense | D | P | 29 | VUS | PM3+PM2+PP3+PP4 | - | Novel |
| 46 | M | *TRPM1* | Exon20 | c.2568G>A | p.Trp856* | Father | Nonsense | - | - | - | Likely pathogenic | PVS1+PM2 | - | PMID:  19896113 |
|  |  | *TRPM1* | Exon4 | c.416G>A | p.G139D | Mother | Missense | D | D | 28.4 | VUS | PM2+PP3 | - | Novel |
| 47 | M | *TRPM1* | Exon21 | c.2750G>A | p.R917H | Father | Missense | D | D | 34 | VUS | PM3+PM2+PP3+PP4 | - | Novel |
|  |  | *TRPM1* | Exon4 | c.416G>A | p.G139D | Mother | Missense | D | D | 28.4 | VUS | PM2+PP3 | - | Novel |
| 48 | M | *TRPM1* | Exon21 | c.2750G>A | p.R917H | Father | Missense | D | D | 34 | VUS | PM3+PM2+PP3+PP4 | - | Novel |
|  |  | *TRPM1* | Exon21 | c.2737G>A | p.G913R | Mother | Missense | D | D | 27.7 | VUS | PM3+PM2+PP3+PP4 | - | Novel |
| 49 | M | *TRPM1* | Exon21 | c.2783G>A | p.R928Q | Father | Missense | D | D | 35 | Likely pathogenic | PM3+PP1+ PM2+PP3+PP4 | - | PMID:  24715752 |
|  |  | *TRPM1* | Exon4 | c.270delA | p.D91Ifs*10 | Mother | Deletion | - | - | - | Pathogenic | PVS1+PM2+PP4 | - | Novel |
| 50 | M | *TRPM1* | Exon21 | c.2855T>C | p.L952R | Father | Missense | D | D | 27.9 | VUS | PM3+PM2+PP3+PP4 | - | Novel |
|  |  | *TRPM1* | Intron22 | c.3061+1G>A |  | Mother | Splicing | - | - | - | Pathogenic | PVS1+PM2+PM3 | DL (0.75) | PMID:  33781268 |
| 51 | F | *TRPM1* | Exon22 | c.2954A>G | p.Q985R | Father | Missense | D | D | 27.1 | VUS | PM3+PM2+PP3+PP4 | - | Novel |
|  |  | *TRPM1* | Exon21 | c.2855T>C | p.L952R | Mother | Missense | D | D | 27.9 | VUS | PM3+PM2+PP3+PP4 | - | Novel |
| 52 | M | *TRPM1* | Intron22 | c.3061+1G>A |  | Mother | Splicing | - | - | - | Pathogenic | PVS1+PM2+PM3 | DL (0.75) | PMID:  33781268 |
|  |  | *TRPM1* | Exon21 | c.2750G>A | p.R917H | Father | Missense | D | D | 34 | VUS | PM3+PM2+PP3+PP4 | - | Novel |
| 53 | M | *TRPM1* | Exon23 | c.3067G>A | p.A1023T | Father | Missense | D | D | 33 | VUS | PM3+PM2+PP3+PP4 | - | Novel |
|  |  | *TRPM1* | Intron22 | c.3061+1G>A |  | Mother | Splicing | - | - | - | Pathogenic | PVS1+PM2+PM3 | DL (0.75) | PMID:  33781268 |
| 54 | F | *TRPM1* | Exon24 | c.3133T>C | p.C1045R | Mother | Missense | D | D | 33 | VUS | PM3+PM2+PP3+PP4 | - | Novel |
|  |  | *TRPM1* | Exon21 | c.2855T>C | p.L952R | Father | Missense | D | D | 27.9 | VUS | PM3+PM2+PP3+PP4 | - | Novel |
| 55 | M | *TRPM1* | Exon24 | c.3208G>A | p.G1070S | Father | Missense | T | P | 28.4 | VUS | PM2+PP4 | - | Novel |
|  |  | *TRPM1* | Intron2 | c.18-3C>G |  | Mother | Splicing | - | - | - | Likely pathogenic | PS3+PM2+PP4 | AL (0.83) | PMID: 20300565 |
| 56 | F | *TRPM1* | Exon21 | c.2737G>A | p.G913R | Mother | Missense | - | - | - | VUS | PM2 | - | Novel |
|  |  | *TRPM1* | Intron16 | c.2022-3C>T |  | Father | Splicing | - | - | - | VUS | PM2 | AG (0.21) | Novel |
| 57 | M | *GRM6* | Exon3 | c.575G>A | p.R192Q | Father | Missense | D | D | 34 | VUS | PM3+PM2+PP3+PP4 | AG (0.96) | PMID:  31106028 |
|  |  | *GRM6* | Exon2 | c.94_162del | p.32-54del | Mother | Deletion | - | - | - | VUS | PM4+PM2+PP4 | - | Novel |
| 58 | M | *GRM6* | Exon3 | c.575G>A | p.R192Q | Father | Missense | D | D | 34 | VUS | PM3+PM2+PP3+PP4 | AG (0.96) | PMID:  31106028 |
|  |  | *GRM6* | Exon2 | c.284T>G | p.L95R | Mother | Missense | D | P | 23.4 | VUS | PM2+PP3+PP4 | - | Novel |
| 59 | M | *GRM6* | Exon9 | c.1639C>T | p.R547C | Father | Missense | D | D | 31 | VUS | PM2+PP3+PP4 | - | Novel |
|  |  | *GRM6* | Exon2 | c.152G>T | p.G51V | Mother | Missense | D | D | 23.9 | VUS | PM3+PM2+PP3+PP4 | - | PMID:  31677249 |

Nucleotide annotation and exons numbering were based on reference sequences NM_022567 (*NYX*), NM_005183 (*CACNA1F*), NM_002420 (*TRPM1*), and NM_000843 (*GRM6*).

Abbreviation: D-disease causing；T-tolerated；B-benign；P-probably damaging; AG-acceptor gain；AL-acceptor loss；DG-donor gain；DL-donor loss；CSNB-congenital stationary night blindness
