# Supplementary material for: Clinical and genetic studies for a cohort of patients with congenital stationary night blindness
Source: Orphanet J Rare Dis. 2024 Mar 6;19:101. doi: 10.1186/s13023-024-03091-3 (PMC10918914; doi:10.1186/s13023-024-03091-3)

Pymol

1. *NYX*: c.611G>T, p.Gly204Val


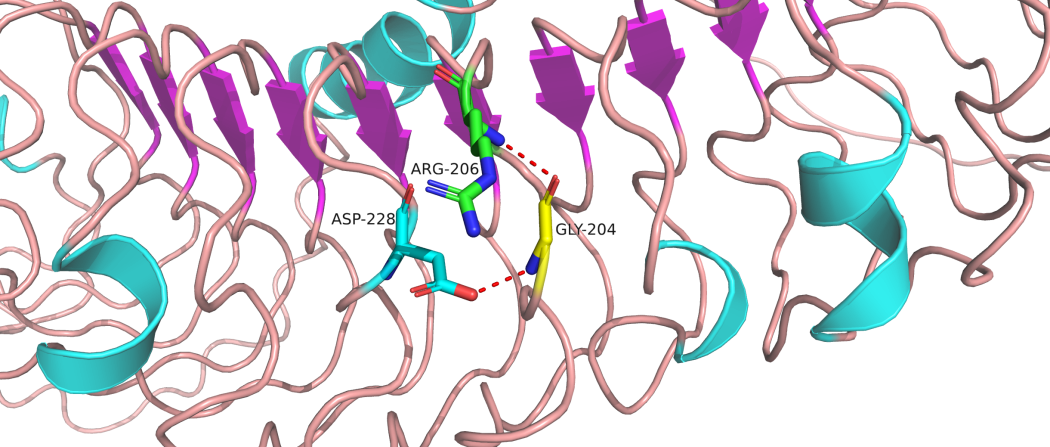
Wild:

Mutant:


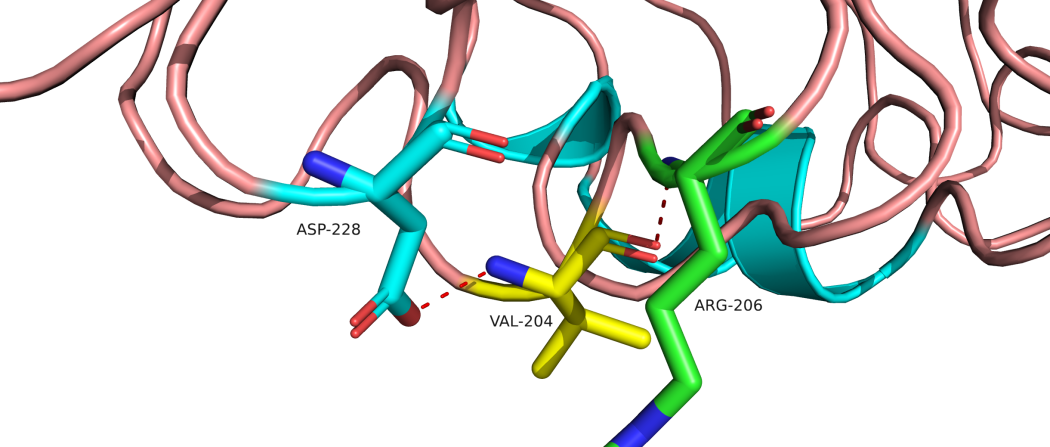


Figure 1. 3-D model construction showed a wild-type amino acid of Glycine was replaced by an amino acid of Valine at codon 204 in NYX.

1. *NYX*: c.662T>G p.Val221Gly

Wild:


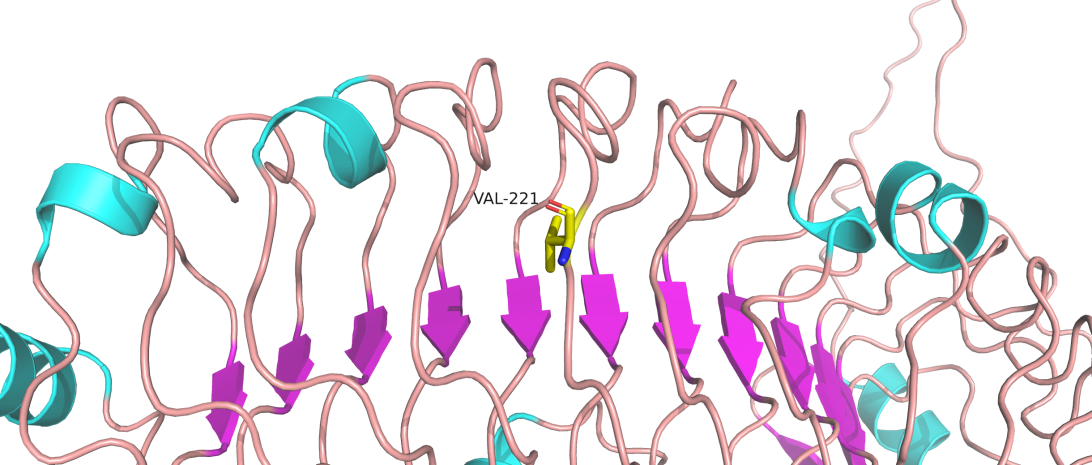


Mutant:

Figure 2. 3-D model construction showed a wild-type amino acid of Valine was replaced by an amino acid of Glycine at codon 221 in NYX.
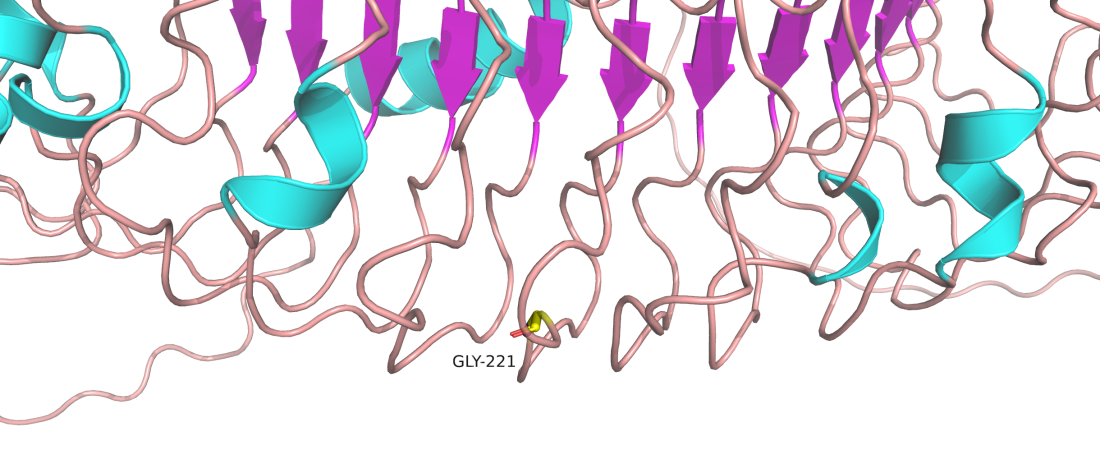


3.NYX: c.719A>G p.Asn240Ser

Wild


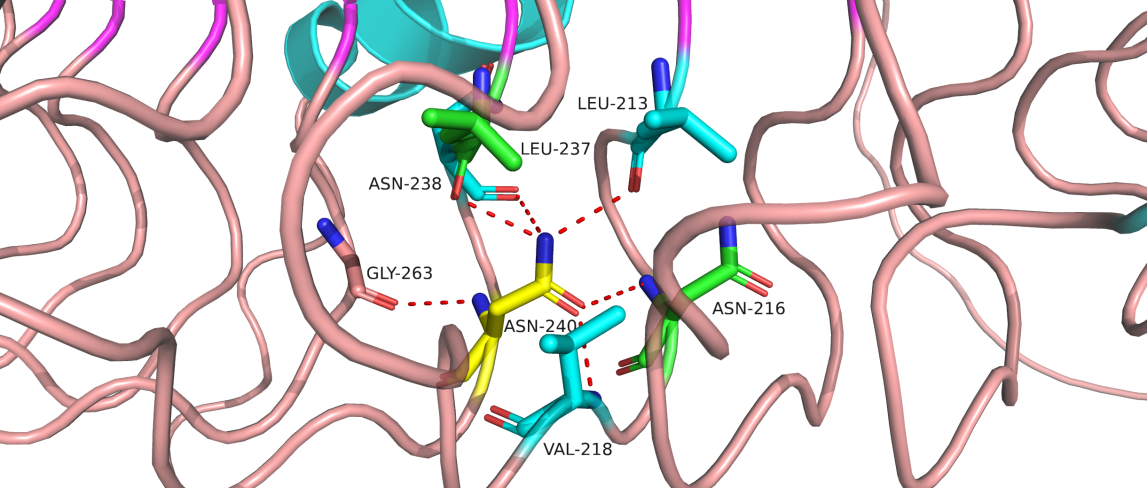


Mutant


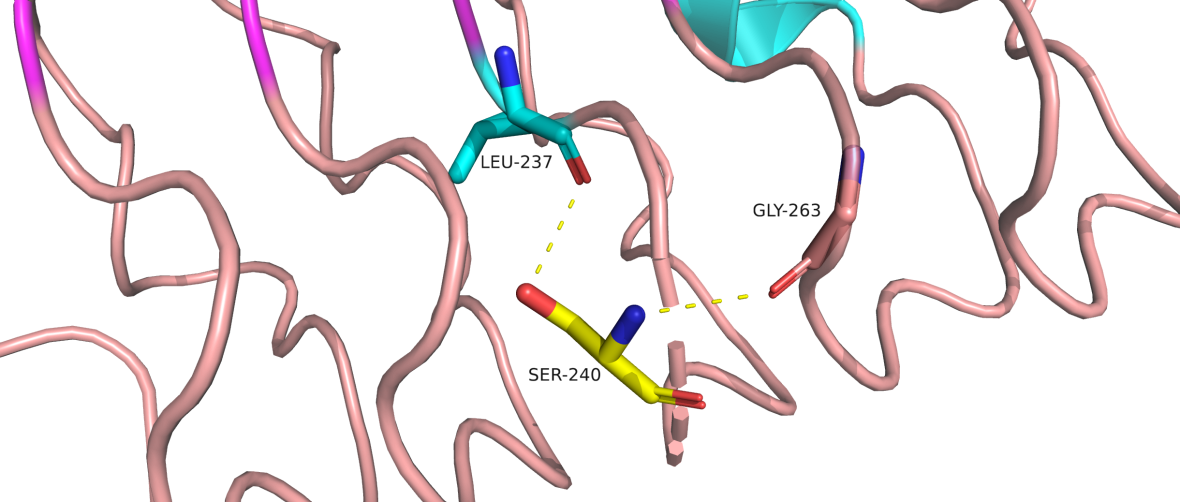


Figure 3. 3-D model construction showed a wild-type amino acid of Asn was replaced by an amino acid of Ser at codon 240 in NYX.

4.CACNA1F : c.1714T>C p.Phe572Leu

Wild


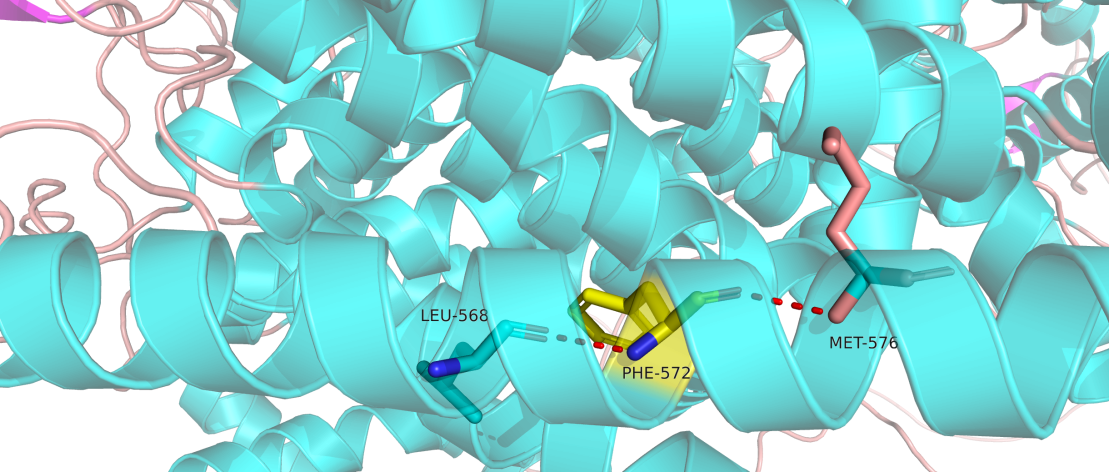


Mutant


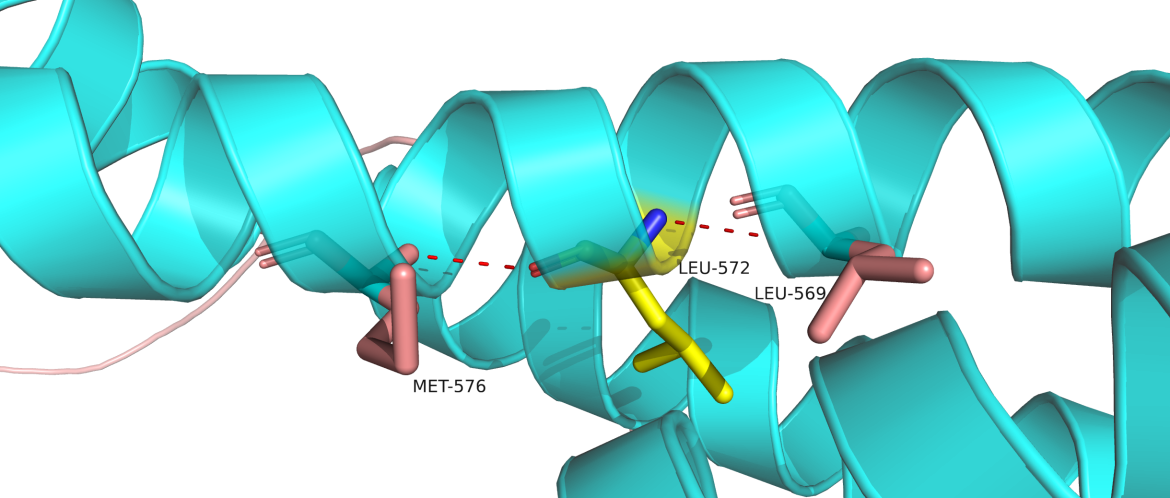


Figure 4. 3-D model construction showed a wild-type amino acid of Phe was replaced by an amino acid of Leu at codon 572 in CACNA1F.

1. CACNA1F： c.2266A>T p.Ile756Phe

Wild


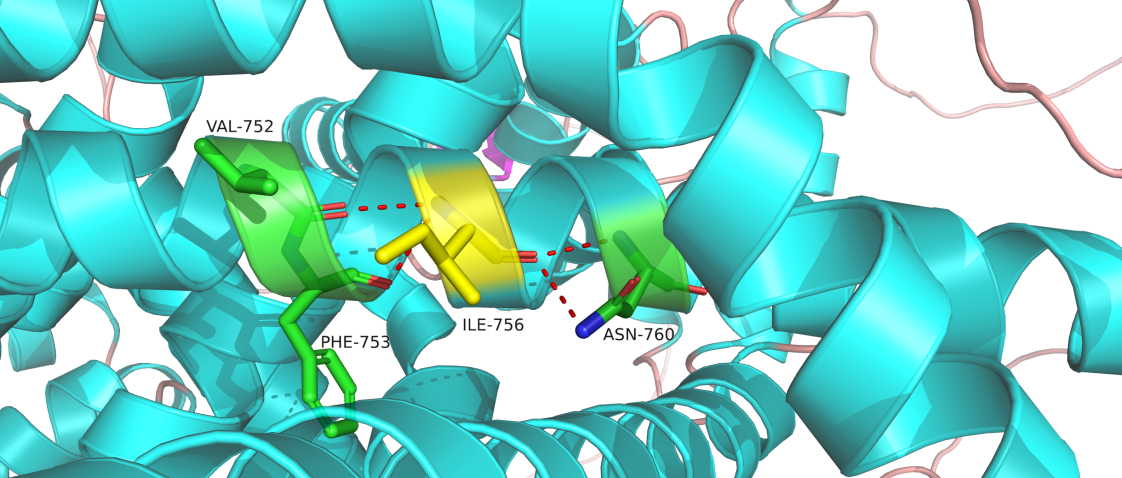


Mutant


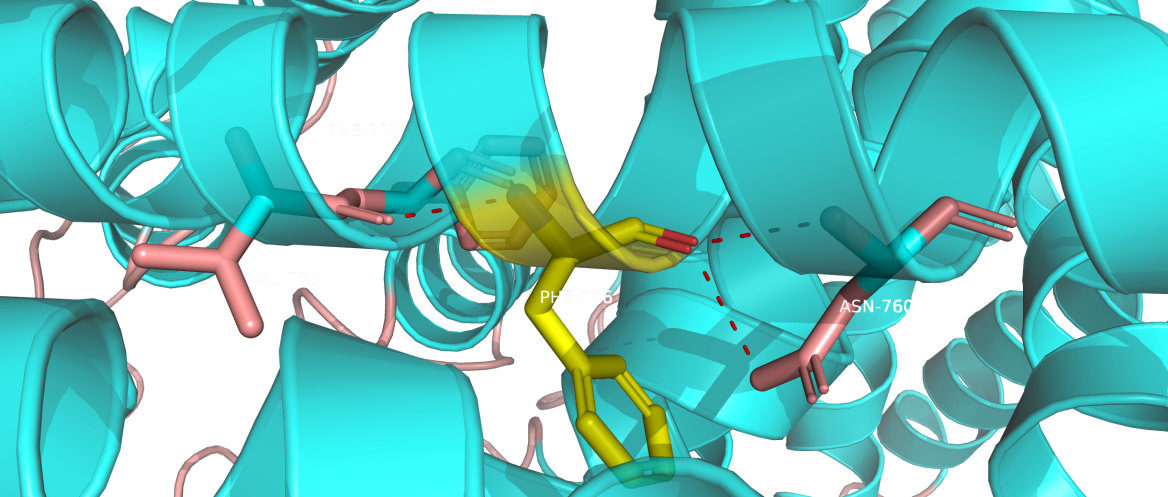


Figure 5. 3-D model construction showed a wild-type amino acid of Ile was replaced by an amino acid of Phe at codon 756 in CACNA1F.

1. CACNA1F ： c.4097T>C p.Phe1366Ser

Wild


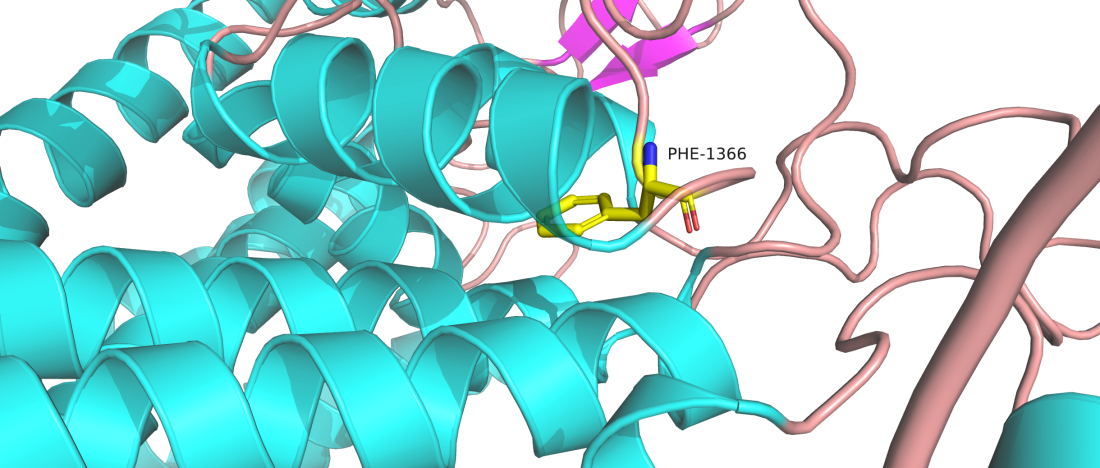


Mutant


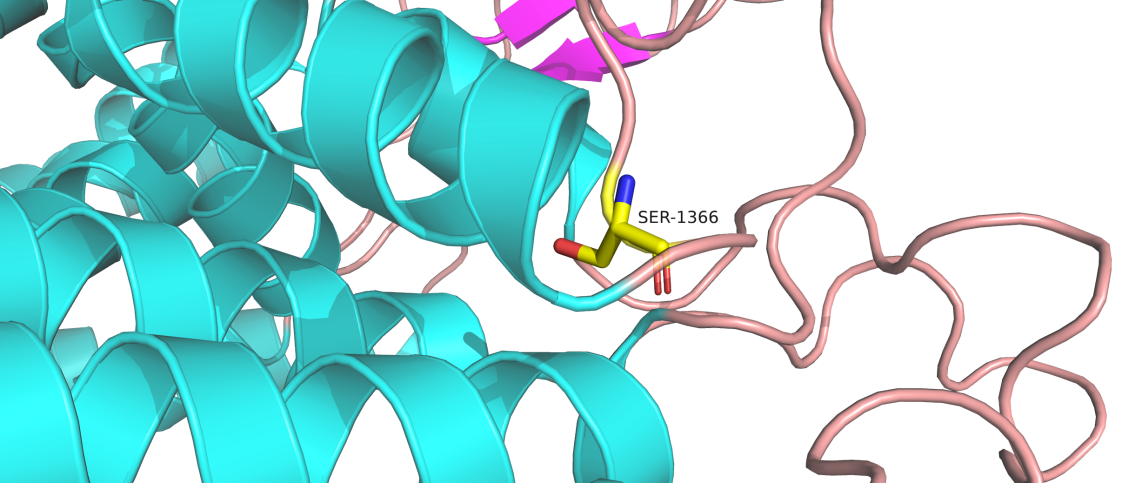


Figure 6. 3-D model construction showed a wild-type amino acid of Phe was replaced by an amino acid of Ser at codon 1366 in CACNA1F.

1. CACNA1F： c.5429G>A p.Arg1810His

Wild


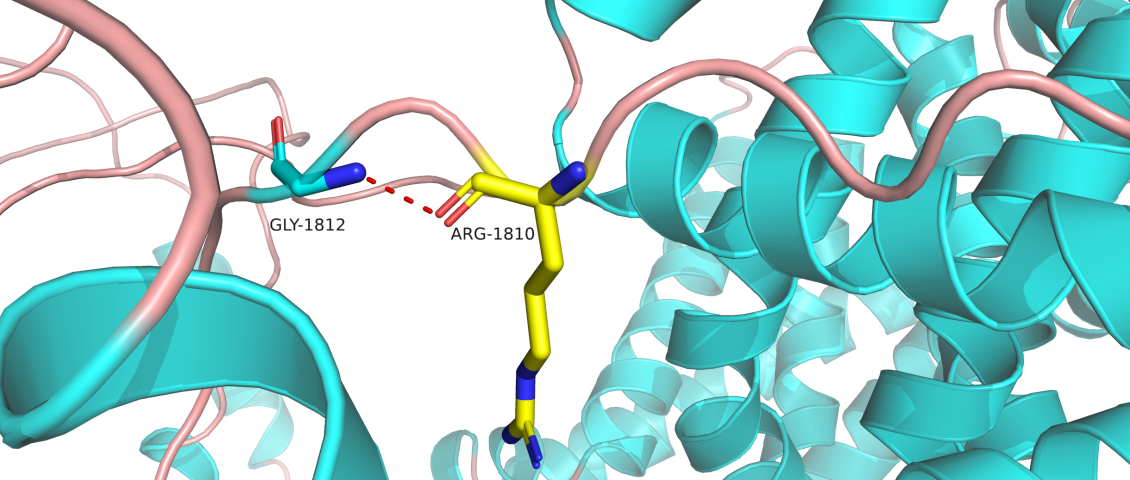


Mutant


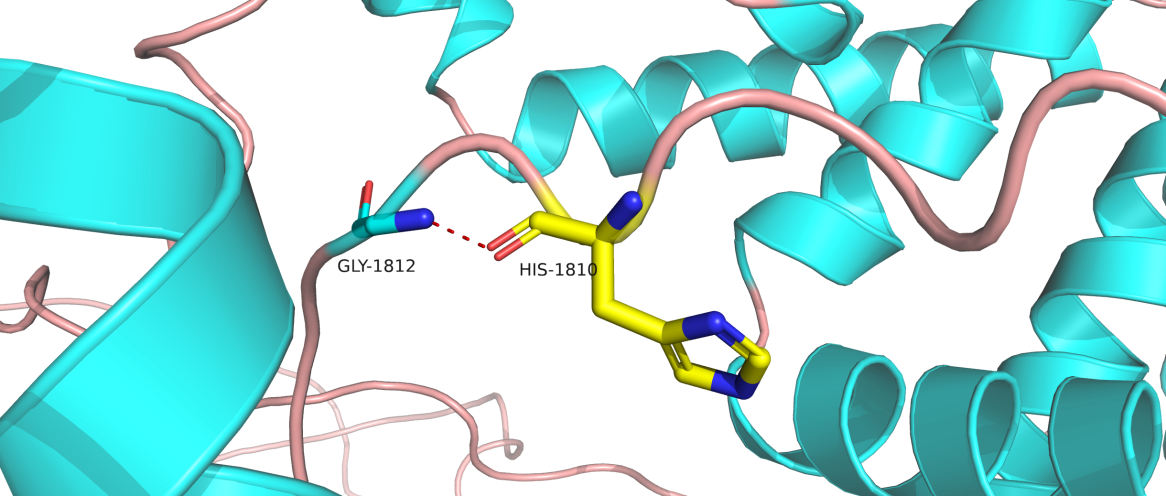


Figure 7. 3-D model construction showed a wild-type amino acid of Arg was replaced by an amino acid of His at codon 1810 in CACNA1F.

1. TRPM1： c.2750G>A p.Arg917His

Wild


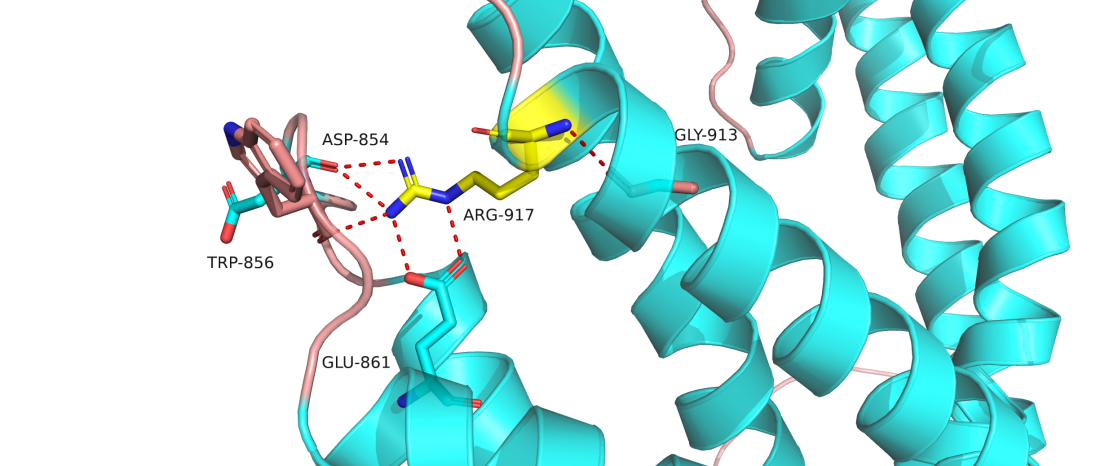
Mutant


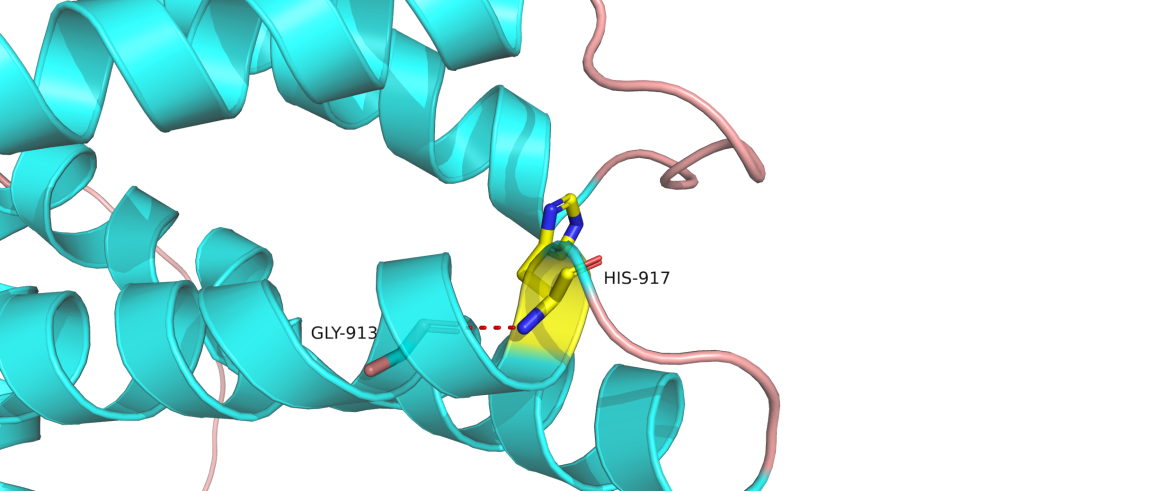


Figure 8. 3-D model construction showed a wild-type amino acid of Arg was replaced by an amino acid of His at codon 917 in TRPM1.

1. TRPM1：c.416G>A p.Gly139Asp

Wild


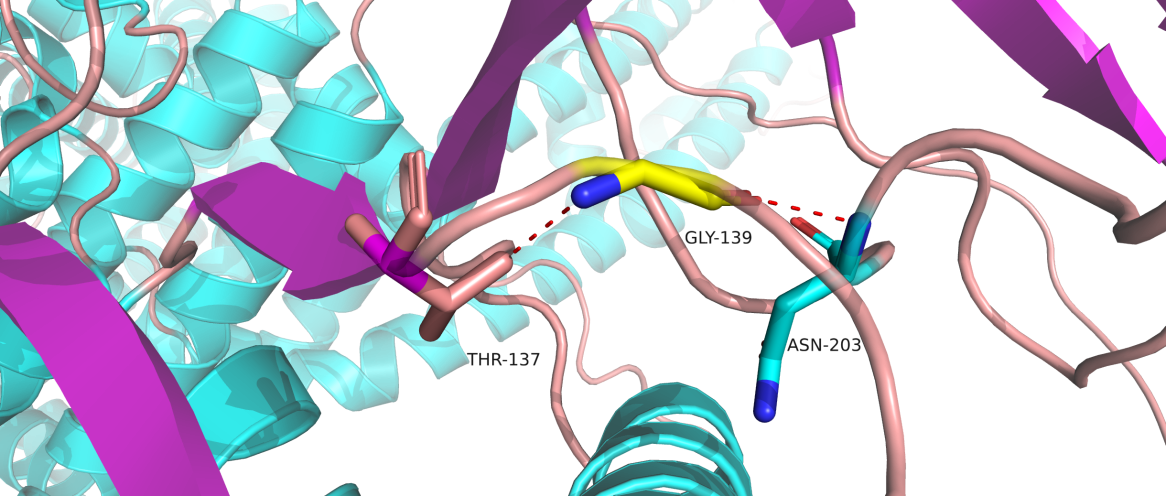
Mutant


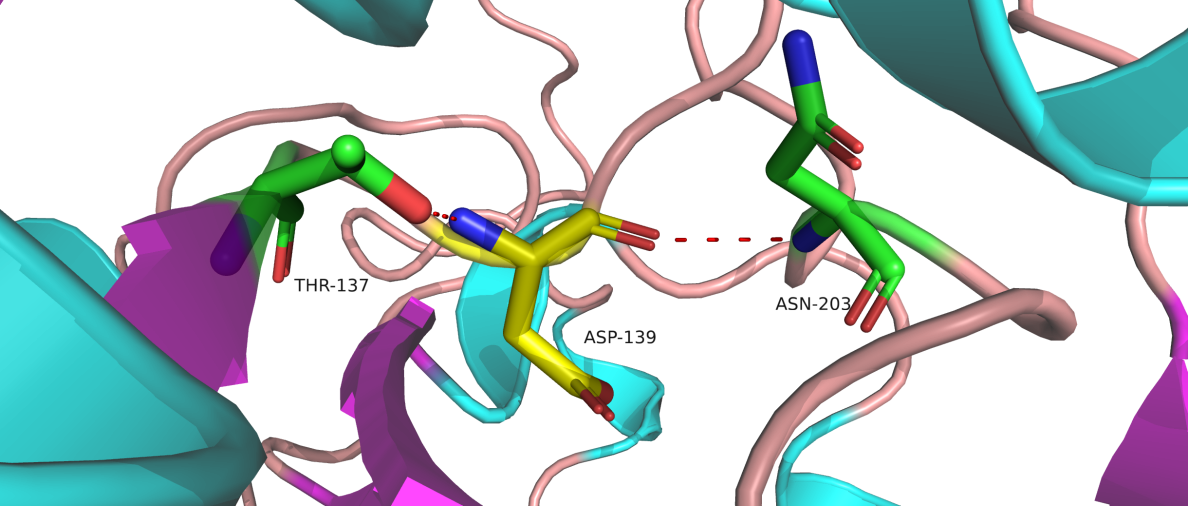


Figure 9. 3-D model construction showed a wild-type amino acid of Gly was replaced by an amino acid of Asp at codon 139 in TRPM1.

1. TRPM1：c.2543T>A p.Val848Asp

Wild


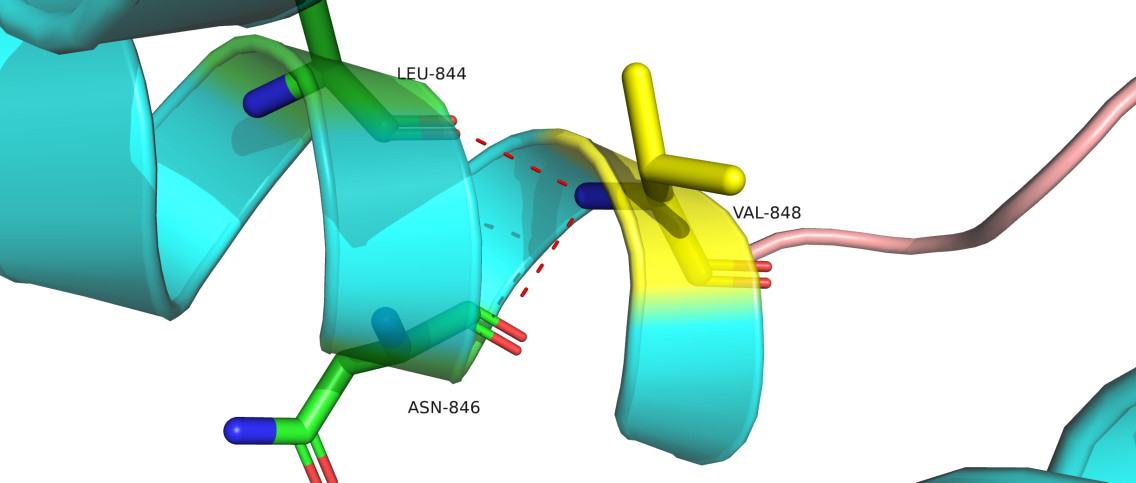


Mutant


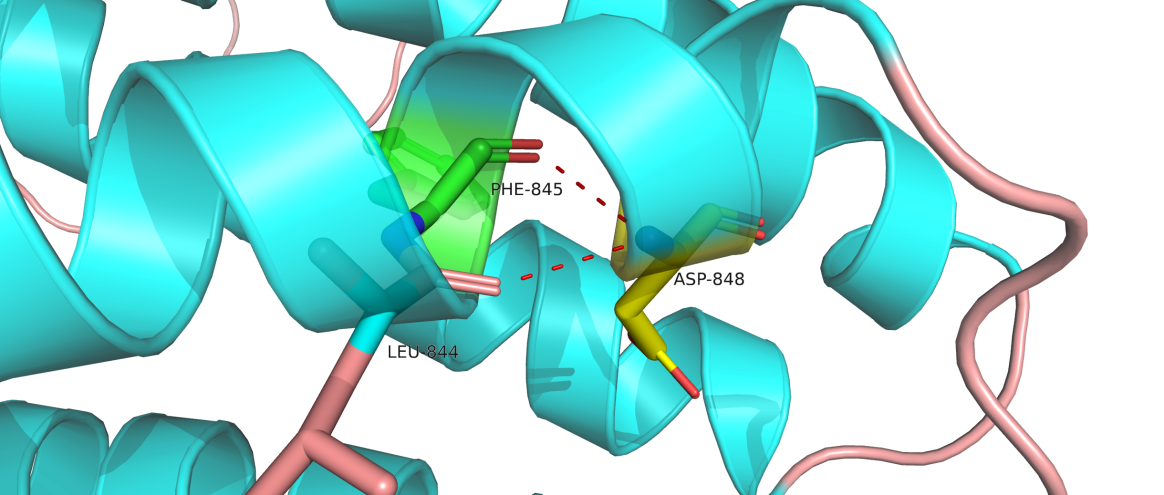


Figure 10. 3-D model construction showed a wild-type amino acid of Val was replaced by an amino acid of Asp at codon 848 in TRPM1.

1. TRPM1： c.2737G>A p.Gly913Arg

Wild


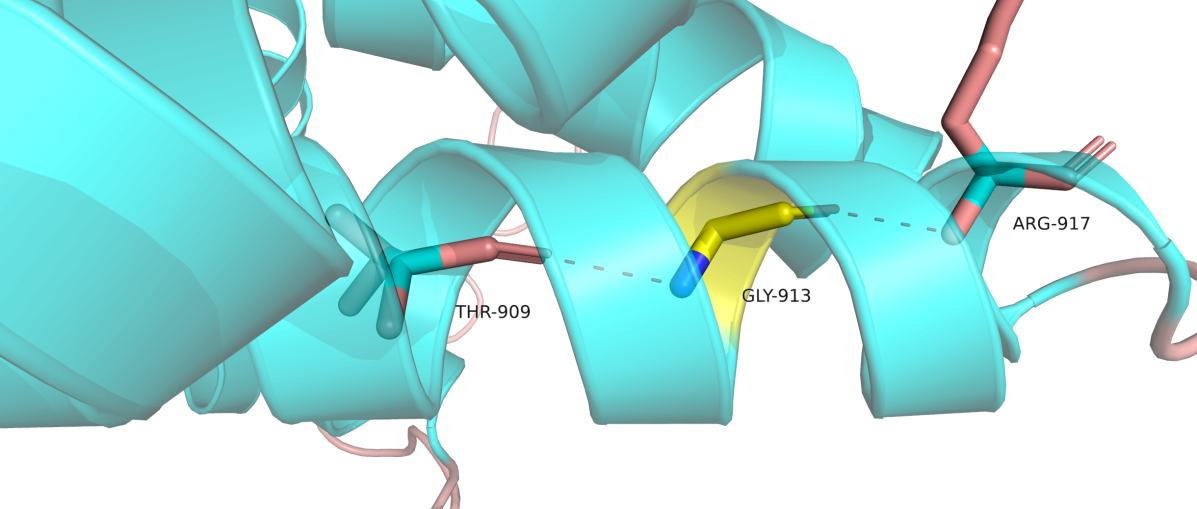
Mutant


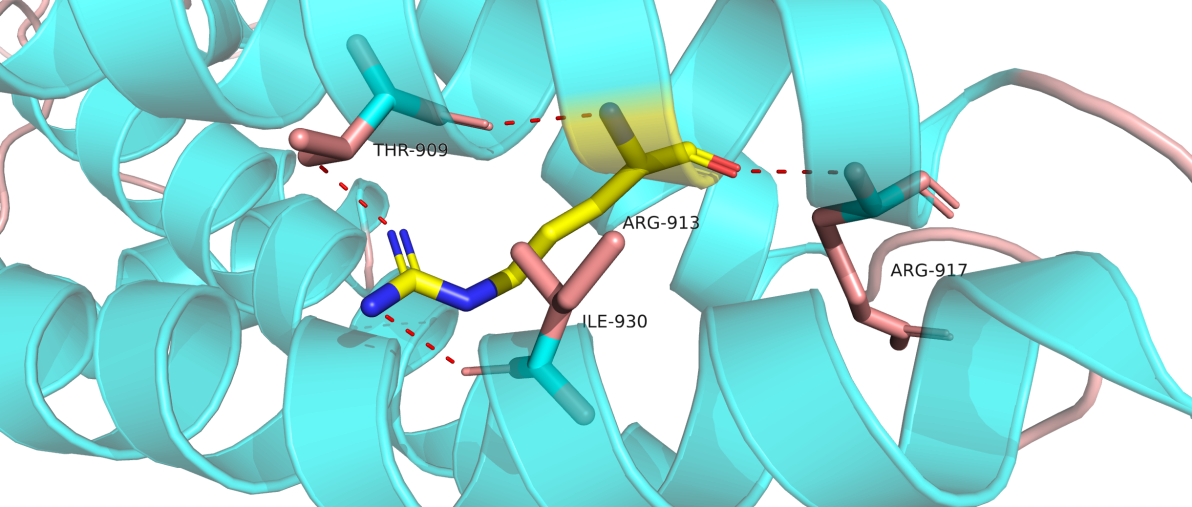


Figure 11. 3-D model construction showed a wild-type amino acid of Gly was replaced by an amino acid of Arg at codon 913 in TRPM1.

1. TRPM1：c.2855T>C p.Leu952Arg

Wild


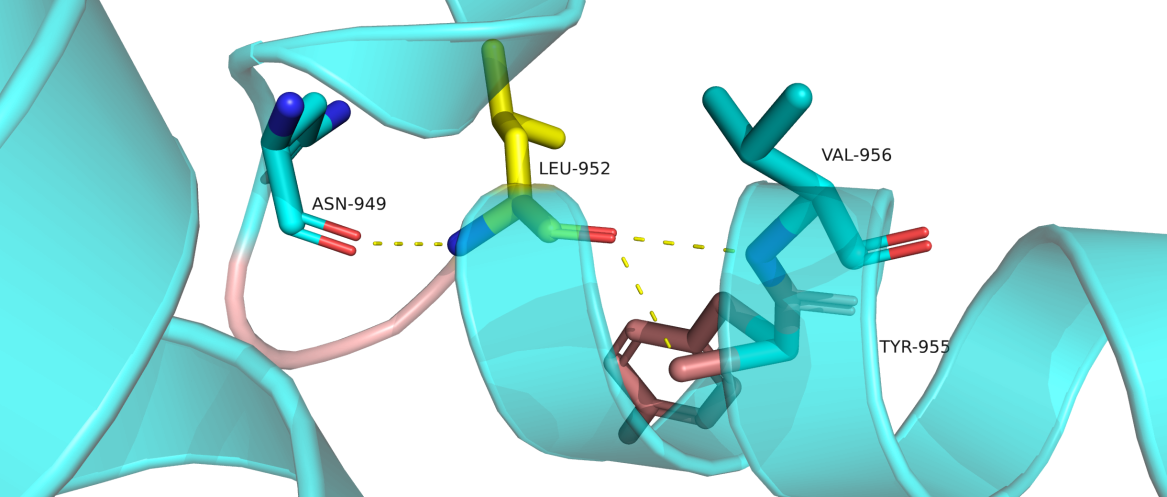


Mutant


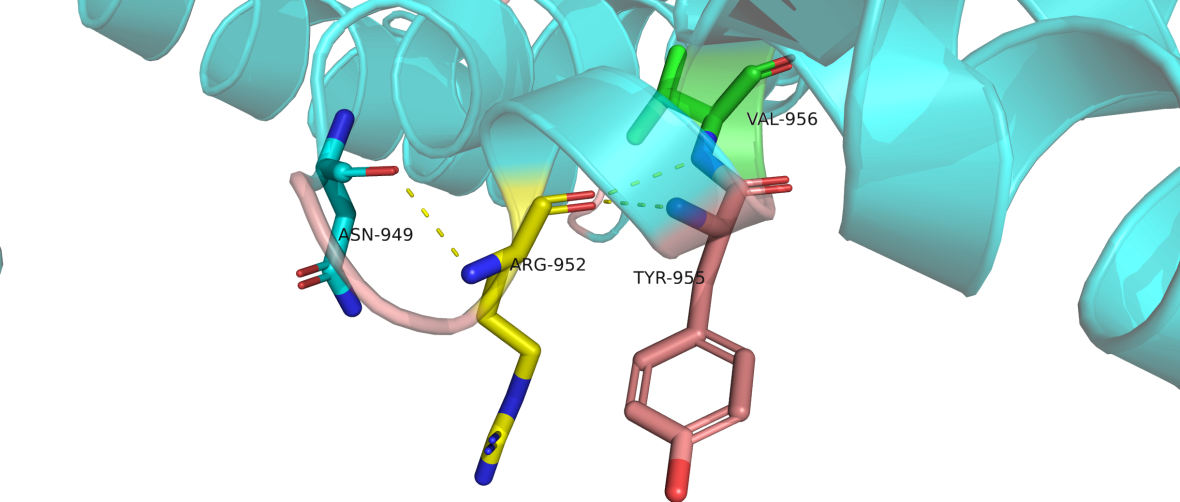


Figure 12. 3-D model construction showed a wild-type amino acid of Leu was replaced by an amino acid of Arg at codon 952 in TRPM1.

1. TRPM1： c.2954A>G p.Gln985Arg

Wild


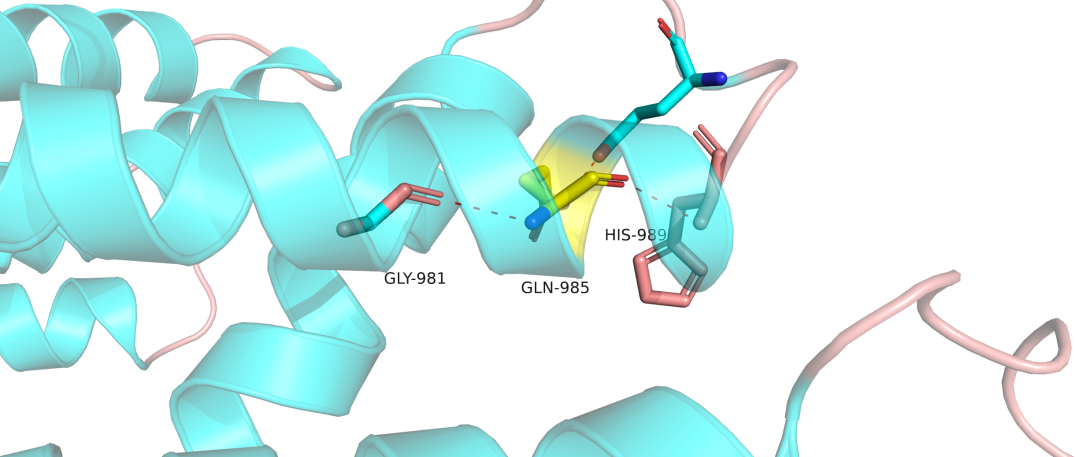
Mutant


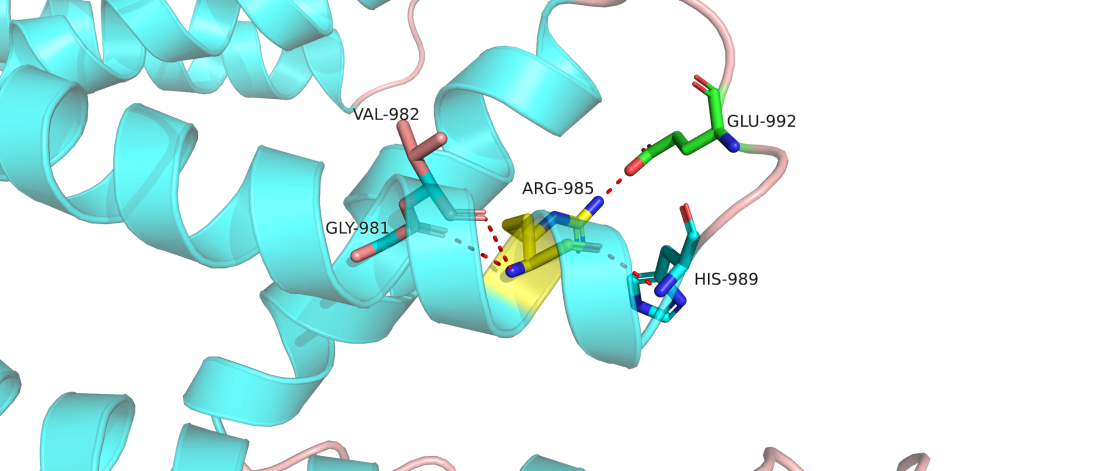


Figure 13. 3-D model construction showed a wild-type amino acid of Gln was replaced by an amino acid of Arg at codon 985 in TRPM1.

1. TRPM1： c.3067G>A P.Ala1023Thr

Wild


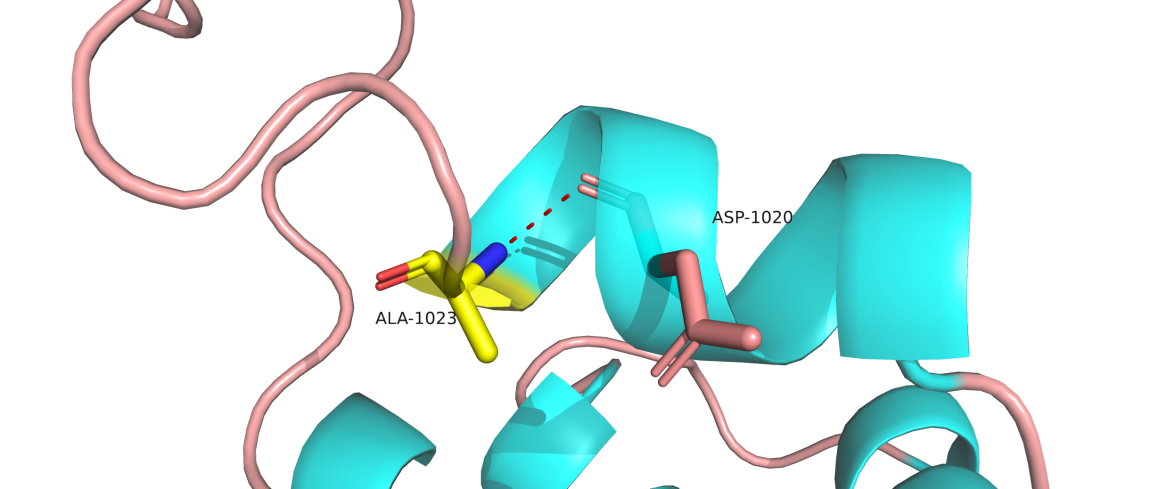
Mutant


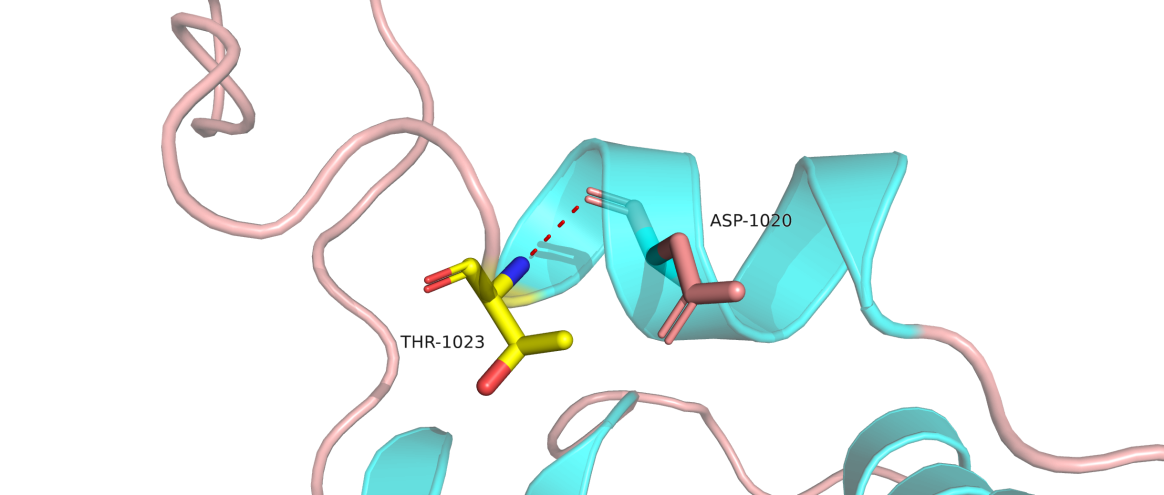


Figure 14. 3-D model construction showed a wild-type amino acid of Ala was replaced by an amino acid of Thr at codon 1023 in TRPM1.

1. TRPM1：c.3133T>C p.Cys1045Arg

Wild


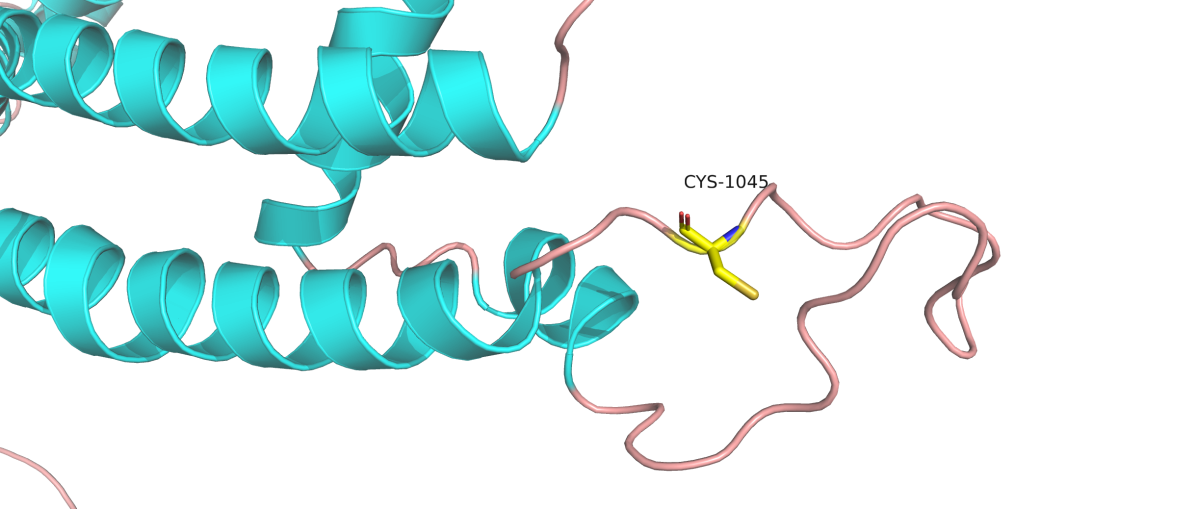


Mutant

Figure 15. 3-D model construction showed a wild-type amino acid of Cys was replaced by an amino acid of Arg at codon 1045 in TRPM1.
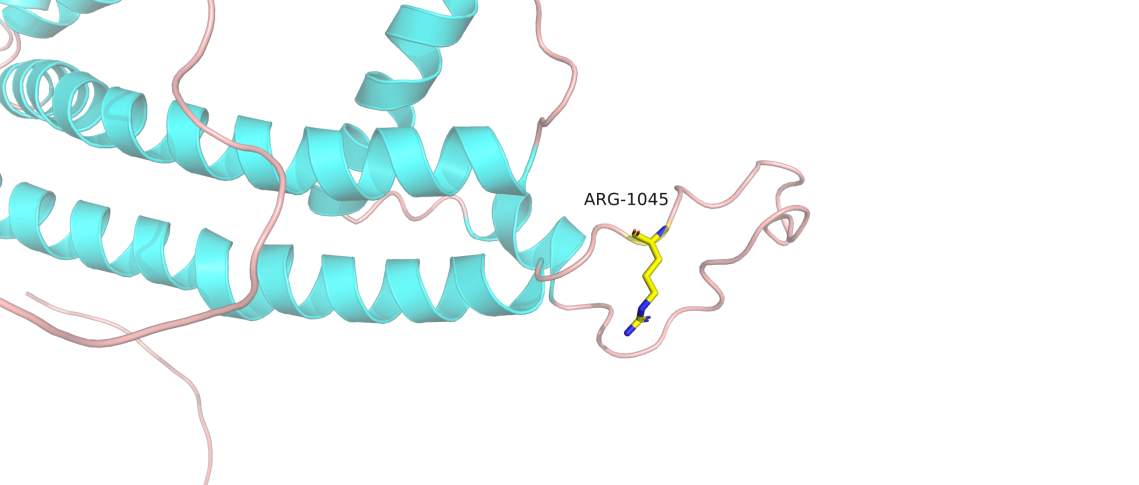

Supplement: Supplementary file 5 — Supplementary Material 5: 3-D structure of protein using the PyMOL program. [file 13023_2024_3091_MOESM5_ESM.docx]
