# Supplementary material for: Clinical and genetic studies for a cohort of patients with congenital stationary night blindness
Source: Orphanet J Rare Dis. 2024 Mar 6;19:101. doi: 10.1186/s13023-024-03091-3 (PMC10918914; doi:10.1186/s13023-024-03091-3)
Supplement: Supplementary file 6 — Supplementary Material 6: Table S1. Novel variations detected in patients with congenital stationary night blindness in this study. [file 13023_2024_3091_MOESM6_ESM.docx]

Table S1. Novel variations detected in patients with congenital stationary night blindness in this study

| **No.** | **No. In S2** | **Gene** | **Location** | **Nucleotide** | **Protein** | **Type** | **SIFT** | **Polyphen-2** | **CADD** | **ACMG** | **Evidence levels** | **SpliceAI** |
| --- | --- | --- | --- | --- | --- | --- | --- | --- | --- | --- | --- | --- |
| 1 | 2 | *NYX* | Exon2 | c.111C>A | p.Cys37* | Nonsense | - | - | - | Pathogenic | PVS1+PM2+PP4 | - |
| 2 | 10 | *NYX* | Exon2 | c.507delC | p.Leu170Cysfs*15 | Deletion | - | - | - | Pathogenic | PVS1+PM2+PP4 | - |
| 3 | 13 | *NYX* | Exon2 | c.611G>T | p.Gly204Val | Missense | D | D | 25.3 | Likely pathogenic | PM2+PP3+PP4 | - |
| 4 | 14 | *NYX* | Exon2 | c.662T>G | p.Val221Gly | Missense | D | D | 23.6 | Likely pathogenic | PM2+PP3+PP4 | - |
| 5 | 15 | *NYX* | Exon2 | c.719A>G | p.Asn240Ser | Missense | D | D | 23.8 | Likely pathogenic | PM2+PP3+PP4 | - |
| 6 | 16 | *NYX* | Exon2 | c.730G>T | p.Glu244* | Nonsense | - | - | - | Pathogenic | PVS1+PM2+PP4 | - |
| 7 | 17 | *NYX* | Exon2 | c.1002delC | p.Arg335Alafs*13 | Deletion | - | - | - | Pathogenic | PVS1+PM2+PP4 | - |
| 8 | 18 | *CACNA1F* | Exon2 | c.144_148delTAAGC | p.Arg50Lysfs*66 | Deletion | - | - | - | Likely pathogenic | PVS1+PM2 | - |
| 9 | 21 | *CACNA1F* | Exon4 | c.527delinsAA | p.Phe176* | Nonsense | - | - | - | Pathogenic | PVS1+PM2+PP4 | - |
| 10 | 24 | *CACNA1F* | Exon13 | c.1536_1537insG | p.Arg513Alafs*37 | Insertion | - | - | - | Pathogenic | PVS1+PS2+PM2 | - |
| 11 | 25 | *CACNA1F* | Exon14 | c.1714T>C | p.Phe572Leu | Missense | D | D | 26.7 | Likely pathogenic | PM2+PP3+PP4 | - |
| 12 | 26 | *CACNA1F* | Exon14 | c.1764T>A | p.Tyr588* | Nonsense | - | - | - | Pathogenic | PVS1+PM2+PP4 | - |
| 13 | 28 | *CACNA1F* | Exon17 | c.2266A>T | p.Ile756Phe | Missense | D | D | 25.8 | Likely pathogenic | PM2+PP3+PM5 | - |
| 14 | 34 | *CACNA1F* | Intron32 | c.3846+5G>C |  | Splicing | - | - | - | VUS | PM2+PP3+PP4 | AG (0.56) |
| 15 | 35 | *CACNA1F* | Exon35 | c.4097T>C | p.Phe1366Ser | Missense | D | D | 27 | Likely pathogenic | PM2+PP3+PP4 | - |
| 16 | 38 | *CACNA1F* | Exon46 | c.5429G>A | p.Arg1810His | Missense | D | B | 34 | VUS | PM2+PP4 | - |
| 17 | 40 | *TRPM1* | Intron16 | c.2022-3C>T |  | Splicing | - | - | - | VUS | PM2 | AG (0.21) |
| 18 | 41 | *TRPM1* | Exon21 | c.2750G>A | p.Arg917His | Missense | D | D | 34 | VUS | PM3+PM2+PP3+PP4 | - |
| 19 | 42 | *TRPM1* | Exon4 | c.416G>A | p.Gly139Asp | Missense | D | D | 28.4 | VUS | PM2+PP3 | - |
| 20 | 43 | *TRPM1* | Exon6 | c.675_676del | p.Arg226Sfs*11 | Deletion | - | - | - | Pathogenic | PVS1+PM2+PP4 | - |
| 21 | 43 | *TRPM1* | Exon20 | c.2685G>A | p.Trp895* | Nonsense | - | - | - | VUS | PM3+PM2+PP3+PP4 | - |
| 22 | 45 | *TRPM1* | Exon16 | c.1896delG | p.W632fs*53 | Deletion | - | - | - | Pathogenic | PVS1+ PM2+PP4 | - |
| 23 | 45 | *TRPM1* | Exon20 | c.2543T>A | p.Val848Asp | Missense | D | P | 29 | VUS | PM3+PM2+PP3+PP4 | - |
| 24 | 48 | *TRPM1* | Exon21 | c.2737G>A | p.Gly913Arg | Missense | D | D | 27.7 | Likely pathogenic | PM3+PM2+PP3+PP4 | - |
| 25 | 49 | *TRPM1* | Exon4 | c.270delA | p.Asp91Ifs*10 | Deletion | - | - | - | Pathogenic | PVS1+PM2+PP4 | - |
| 26 | 50 | *TRPM1* | Exon21 | c.2855T>C | p.Leu952Arg | Missense | D | D | 27.9 | Pathogenic | PS4+PM3+PM2+PP3+PP4 | - |
| 27 | 51 | *TRPM1* | Exon22 | c.2954A>G | p.Gln985Arg | Missense | D | D | 27.1 | Likely pathogenic | PM3+PM2+PP3+PP4 | - |
| 28 | 53 | *TRPM1* | Exon23 | c.3067G>A | P.Ala1023Thr | Missense | D | D | 33 | VUS | PM3+PM2+PP3+PP4 | - |
| 29 | 54 | *TRPM1* | Exon24 | c.3133T>C | p.Cys1045Arg | Missense | D | D | 33 | VUS | PM3+PM2+PP3+PP4 | - |
| 30 | 55 | *TRPM1* | Exon24 | c.3208G>A | p.Gly1070Ser | Missense | T | P | 28.4 | VUS | PM2+PP4 | - |
| 31 | 58 | *GRM6* | Exon2 | c.284T>G | p.Leu95Arg | Missense | D | P | 23.4 | VUS | PM2+PP3+PP4 | - |
| 32 | 59 | *GRM6* | Exon9 | c.1639C>T | p.Arg547Cys | Missense | D | D | 31 | VUS | PM2+PP3+PP4 | - |

Nucleotide annotation and exons numbering were based on reference sequences NM_022567 (*NYX*), NM_005183 (*CACNA1F*), NM_002420 (*TRPM1*), and NM_000843 (*GRM6*).

Abbreviation: D - disease causing; T - tolerated; B - benign; P - probably damaging; AG - acceptor gain; AL- acceptor loss; DG - donor gain; DL- donor loss.
